# Iridium Corroles Exhibit Weak Near-Infrared Phosphorescence but Efficiently Sensitize Singlet Oxygen Formation

**DOI:** 10.1038/s41598-020-64389-3

**Published:** 2020-05-05

**Authors:** Ivar K. Thomassen, Laura J. McCormick-McPherson, Sergey M. Borisov, Abhik Ghosh

**Affiliations:** 1 Department of Chemistry, UiT – The Arctic University of Norway, N-9037 Tromsø, Norway; 2 Advanced Light Source, Lawrence Berkeley National Laboratory, Berkeley, California, 94720-8229 United States; 3 Institute of Analytical Chemistry and Food Chemistry, Graz University of Technology, Stremayrgasse 9, 8010 Graz, Austria

**Keywords:** Chemistry, Materials science, Optics and photonics

## Abstract

Six-coordinate iridium(III) triarylcorrole derivatives, Ir[T*p*XPC)]L_2_, where T*p*XPC = tris(*para*-X-phenyl)corrole (X = CF_3_, H, Me, and OCH_3_) and L = pyridine (py), trimethylamine (tma), isoquinoline (isoq), 4-dimethylaminopyridine (dmap), and 4-picolinic acid (4pa), have been examined, with a view to identifying axial ligands most conducive to near-infrared phosphorescence. Disappointingly, the phosphorescence quantum yield invariably turned out to be very low, about 0.02 – 0.04% at ambient temperature, with about a two-fold increase at 77 K. Phosphorescence decay times were found to be around ~5 µs at 295 K and ~10 µs at 77 K. Fortunately, two of the Ir[T*p*CF_3_PC)]L_2_ derivatives, which were tested for their ability to sensitize singlet oxygen formation, were found to do so efficiently with quantum yields Φ(^1^O_2_) = 0.71 and 0.38 for L = py and 4pa, respectively. Iridium corroles thus may hold promise as photosensitizers in photodynamic therapy (PDT). The possibility of varying the axial ligand and of attaching biotargeting groups at the axial positions makes iridium corroles particularly exciting as PDT drug candidates.

## Introduction

The 5d transition metal corroles represent an unusual class of size-mismatched metal-ligand assemblies, which combine a large 5d ion and a sterically constrained, macrocyclic corrole ligand^1^. Although many of the complexes were initially synthesized as part of curiosity-driven exercises, their photophysical properties now promise a wide range of practical applications^2,3^, as near-IR emitters, as oxygen sensors, and as photosensitizers for photodynamic therapy, dye-sensitized solar cells, and triplet-triplet annihilation upconversion^4–9^. Iridium corroles were among the first 5d metallocorroles to be synthesized^10^ and reported as exhibiting as near-IR phosphorescence at room temperature^4,5^. Subsequently, Au^11–14^, OsN^15^, ReO^16^, and Pt^17^ corroles were synthesized and found to exhibit significantly stronger phosphorescence^6–9^. In this reexamination of six-coordinate Ir corroles (structures depicted below), we attempted to determine whether different axial ligands, including pyridine (py), trimethylamine (tma), isoquinoline (isoq), 4-dimethylaminopyridine (dmap), and 4-picolinic acid (4pa), might be exploited to enhance the phosphorescent behavior.

## Results and Discussion

### Early spectroscopic and structural studies

**Figure 1:**
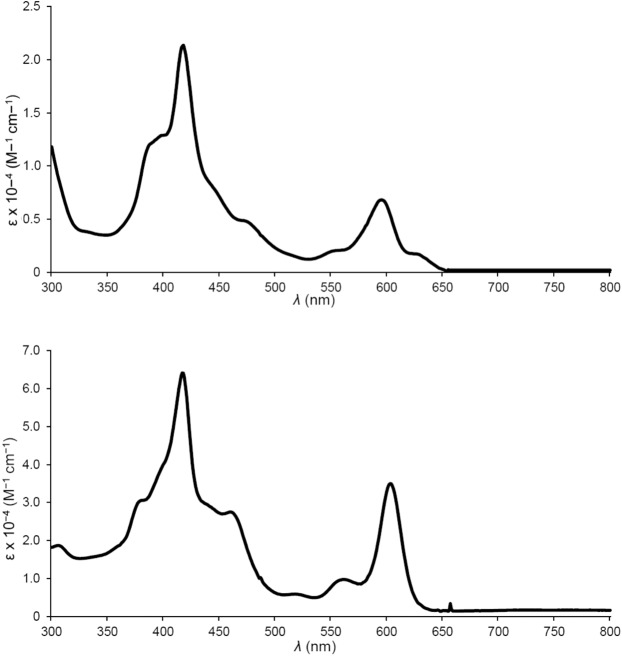
Representative UV-vis spectra in dichloromethane: Ir[(T*p*MePC)]tma_2_ (top) and Ir[(T*p*MePC)]py_2_ (bottom).

**Figure 2:**
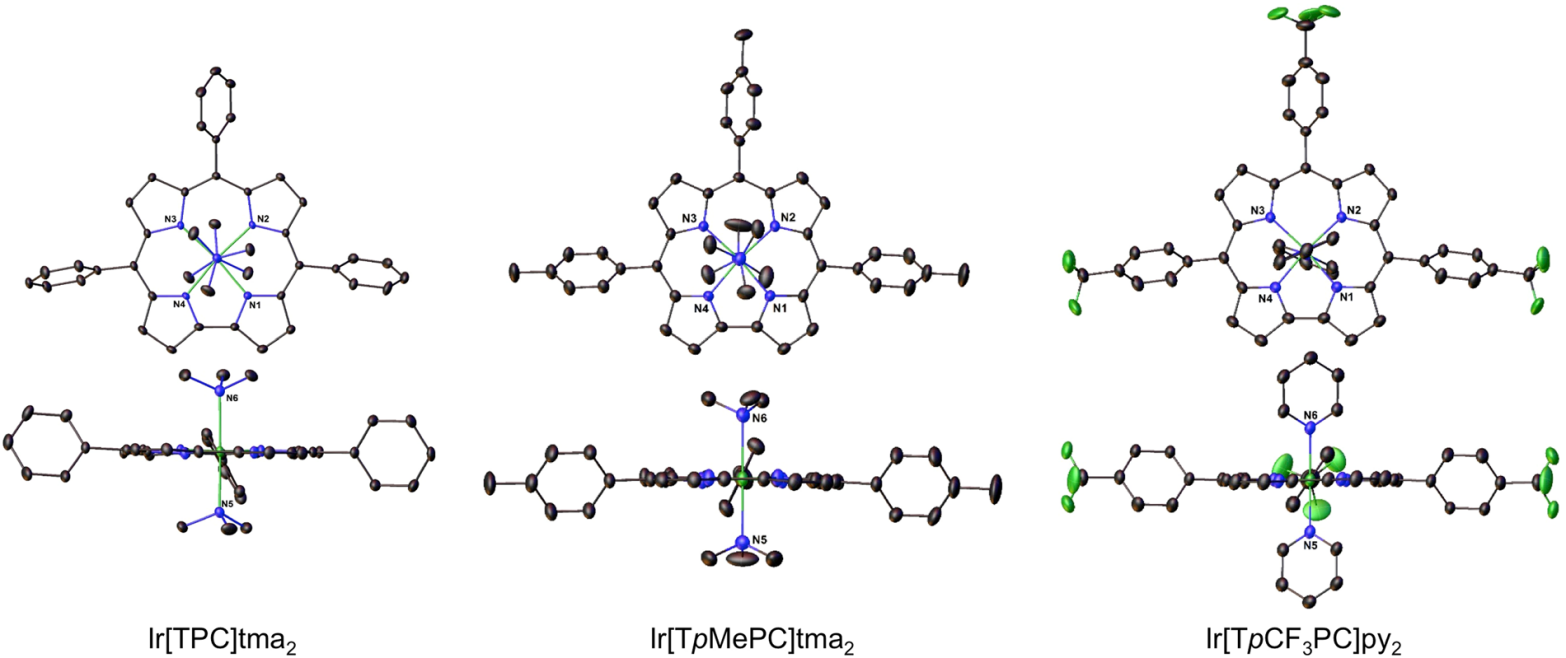
X-ray structures (top and side views) of Ir[TPC]tma_2_ (left), Ir[T*p*MePC]tma_2_ (middle), and Ir[T*p*CF_3_PC]py_2_ (right). Bond distances (Å) for Ir[TPC]tma_2_: Ir-N1 1.9543(16), Ir-N2 1.9728(16), Ir-N3 1.9745(16), Ir-N4 1.9489(17), Ir-N5 2.1795(18), Ir-N6 2.1672(18). Bond distances (Å) for Ir[T*p*MePC]tma_2_. Ir-N1 1.953(2), Ir-N2 1.9746(19), Ir-N3 1.978(2), Ir-N4 1.948(2), Ir-N5 2.178(3), Ir-N6 2.176(3). Bond distances (Å) for Ir[T*p*CF_3_PC]py_2_: Ir-N1 1.946(2), Ir-N2 1.9702(18), Ir-N5 2.055(2); note that N5 and N6 are crystallographically equivalent, as are N2 and N3, and N1 and N4.

Four different *meso*-tris(*para*-X-phenyl)corrole ligands, H_3_[T*p*XPC] (X = CF_3_, H, Me, and OMe), and two different axial ligands, pyridine (py) and trimethylamine (tma), were initially investigated. The compounds were all found to exhibit broad, asymmetric Soret bands with a maximum at 416 ± 2 nm and intense Q bands with the maximum varying over 600 ± 5 nm (Fig. 1 and Table 1). Three of the complexes – Ir[TPC]tma_2_, Ir[T*p*MePC]tma_2_, and Ir[T*p*CF_3_PC]py_2_ (Fig. 2, Table 2) – could be structurally characterized, revealing planar metallocorrole macrocycles with Ir-N_corrole_ distances of 1.95-1.97 Å and Ir-N_axial_ distances of 2.17-2.18 Å. Electrochemical studies revealed two reversible oxidation potentials, including a rather low first oxidation potential that ranged from 0.2 to 0.44 V vs. the saturated calomel electrode (Table 1). In general, a reduction potential could not be observed within the available potential window for the solvent (dichloromethane), except for Ir[T*p*CF_3_PC]py_2_, which showed a reduction potential of -1.71 V, a reflection of the highly electron-rich character of the macrocycle in Ir corroles (Table 1). All these properties are qualitatively consistent with those observed for Ir tris(pentafluorophenyl)corrole derivatives^10^.

**Table 1:** Absorption maxima (λ, nm) and redox potentials (V vs. SCE) for Ir(III) corroles in dichloromethane^a^.

Complex	B	Q	*E*_½ox1_	*E*_½ox2_	*E*_½red1_
Ir[TPC]tma_2_	384, 416^*^, 468	596^*^	0.31	1.09	—
Ir[TPC]py_2_	414^*^, 459	562, 602^*^	0.28	1.00	—
Ir[T*p*MePC]tma_2_	384, 417^*^, 468	598^*^	0.26	1.02	—
Ir[T*p*MePC]py_2_	417^*^, 460	562, 603^*^	0.52	1.02	—
Ir[T*p*OMePC]tma_2_	384, 417^*^, 468	600^*^	0.23	0.94	—
Ir[T*p*OMePC]py_2_	416^*^, 458	563, 605^*^	0.20	0.89	—
Ir[T*p*CF_3_PC]tma_2_	418^*^	595^*^	0.44	1.18	—
Ir[T*p*CF_3_PC]py_2_	416^*^	602^*^	0.41	1.11	-1.71
Ir[T*p*CF_3_PC]dmap_2_	418^*^	606^*^	—	—	—
Ir[T*p*CF_3_PC]4pa_2_	414^*^	602^*^	—	—	—
Ir[T*p*CF_3_PC]isoq_2_	417^*^	603^*^	—	—	—

^a^Asterisks indicate the most intense peaks in the Soret and Q regions.

**Table 2:** Crystallographic data for six-coordinate Ir(III) corroles.

Compound	Ir^III^[TPC]tma_2_	Ir^III^[T*p*MePC]tma_2_	Ir^III^[T*p*CF_3_PC]py_2_
Chemical formula	C_43_H_41_IrN_6_	C_54.25_H_66.25_IrN_6_	C_50_H_30_F_9_IrN_6_
Formula mass	834.02	994.58	1078.00
Crystal system	Monoclinic	Monoclinic	Monoclinic
Space group	*P* 2_1_/*n*	*P* 2_1_/*n*	*C* 2/*c*
*λ* [Å]	0.7749	0.7293	0.7749
*a* [Å]	11.4814(5)	12.8383(7)	18.1543(9)
*b* [Å]	21.7131(10)	15.3802(8)	16.8434(7)
*c* [Å]	13.7137(6)	24.6017(13)	14.2251(7)
*α* [^o^]	90	90	90
*β* [^o^]	94.371(2)	92.934(2)	112.321(2)
*ϒ* [^o^]	90	90	90
*Z*	4	4	4
V [Å]	3408.8(3)	4851.4(4)	4023.8(3)
Temperature [K]	100(2)	150(2)	100(2)
Density [g cm^-3^]	1.625	1.362	1.779
Meas. reflections	46872	91185	40581
Unique reflections	10433	16047	10326
Parameters	457	612	324
Restraints	0	52	6
*R*_int_	0.0478	0.0599	0.0431
*θ* range [^o^]	1.919-33.659	1.603-32.374	1.867-41.256
*R*_*1*_, *wR*_*2*_ all data	0.0276, 0.0551	0.0407, 0.1113	0.0463, 0.0935
*S* (GooF) all data	1.049	0.828	1.039
Max/min res. dens. [e.Å^-3^]	1.123/-1.092	0.903/-1.735	3.436/-2.876

### Near-IR phosphorescence

**Figure 3:**
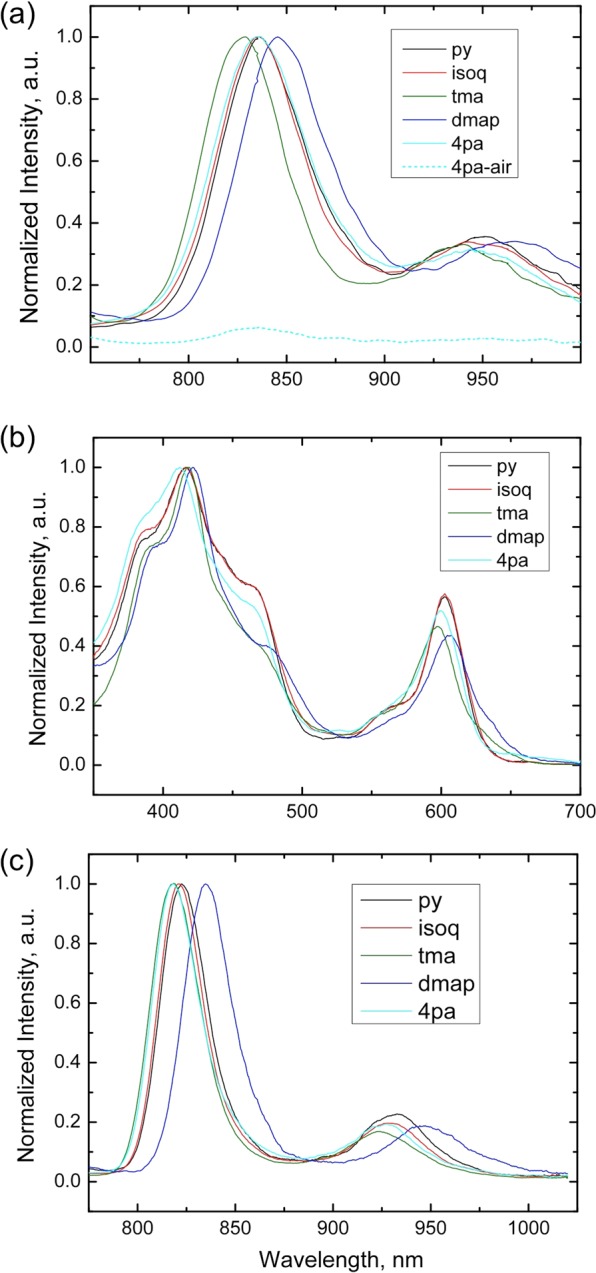
Photophysical properties of Ir-T*p*CF_3_PC complexes with the axial ligands indicated. (**a**) Emission spectra in anoxic toluene (unless otherwise mentioned) at 295  K with λ_ex_ = 595-605  nm (at the maximum of the Q band). The bis-4pa complex was measured in EtOH (anoxic conditions – solid line, air-saturated solvent – dashed line). (**b**) Excitation spectra at 295  K in the same solvents as in (**a**). The emission was detected in the maximum of the emission spectra. RG9 long-pass filter was installed in the emission channel to eliminate the monochromator artefacts. (**c**) Emission spectra in frozen glasses (77 K) with λ_ex_ = 595-605  nm (at the maximum of the Q-band). The bis-4pa complex was measured in 4:1 v/v ethanol:methanol, while the other complexes were measured in 2:3 v/v toluene:tetrahydrofuran.

**Figure 4:**
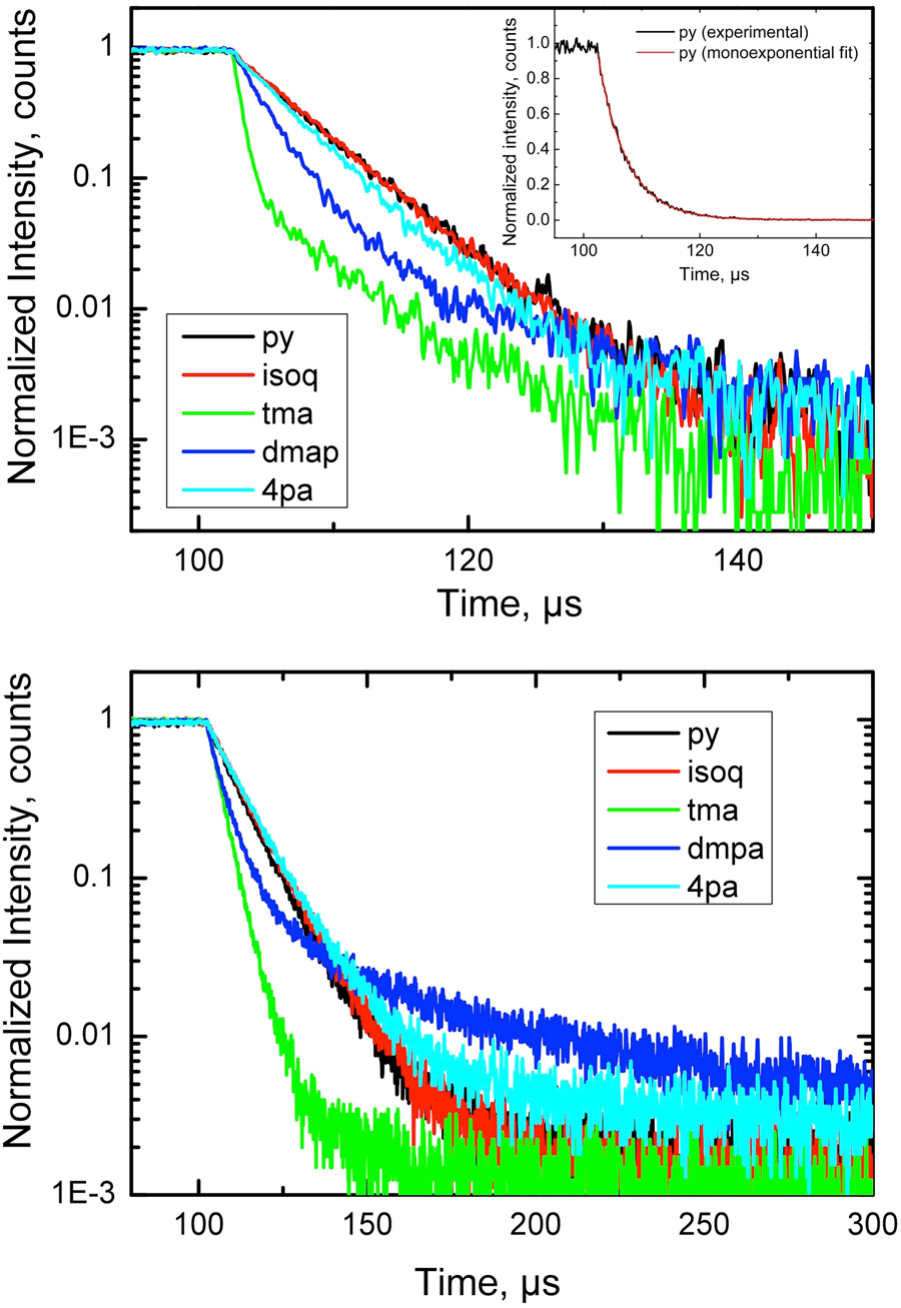
Logarithmic plots for the phosphorescence decay of Ir-T*p*CF_3_PC complexes in anoxic solutions at 295 K (above) and in frozen glasses at 77 K (below). The inset depicts the phosphorescence decay of the pyridine complex and the fit according to a monoexponential decay model. The solvents are the same as in Fig. 3.

For the final set of measurements, we chose to focus on the T*p*CF_3_PC derivatives but with an expanded set of axial ligands (L) including py, tma, isoquinoline (isoq), 4-dimethylaminopyridine (dmap), and 4-picolinic acid (4pa). All the complexes were found to exhibit weak phosphorescence in the near-infrared part of the spectrum in anoxic solutions (Fig. 3a). The phosphorescence was almost completely quenched in the presence of molecular oxygen. The excitation spectra (Fig. 3b) matched the absorption spectra very well. As expected, the emission spectra were much narrower at 77 K (Fig. 3c), which enabled more precise determination of the triplet state energies from the edge of the emission spectra. Disappointingly, the phosphorescence quantum yields for all the complexes turned out to be very low, which made their precise estimation difficult. Quantum yields of around 0.02 – 0.04% were estimated at ambient temperature with about a two-fold increase at 77 K. The phosphorescence of the bis-pyridine and bis-isoquinoline complexes was found to be stronger than that of bis-tma and bis-dmap complexes. Furthermore, the emission spectra for the pyridine, isoquinoline, and 4-picolinic acid complexes turned out almost identical (Fig. 3a, Table 3). The decay time profiles for these complexes are mono-exponential (Fig. 4). The fits provide very similar values of ~5 µs at 295 K and ~10 µs at 77 K. Interestingly, in the case of the bis-dmap complex, a relatively long-decaying component was observed both at ambient temperature and at 77 K.

**Table 3:** Photophysical properties of Ir[T*p*CF_3_PC)]L_2_ derivatives^a–e^.

Complex	295 K^a^			77K^c^			
	λ_max,em_ (nm)	τ (µs)	QY (%)	λ_max,em_ (nm)	τ (µs)	QY (%)	E_T_^e^, cm^−1^
Ir[T*p*CF_3_PC)]py_2_	836	5.6	∼0.04	823	9.8	∼0.04	12560
Ir[T*p*CF_3_PC]isoq_2_	836	4.9	∼0.04	821	10.4	∼0.06	12560
Ir[T*p*CF_3_PC]tma_2_	828	0.6 (38%); 5.1 (62%)	∼0.02	818	4.2	∼0.06	12630
Ir[T*p*CF_3_PC]dmap_2_	846	2.3 (78%); 8.1 (22%)	∼0.02	835	5.2 (61%); 36 (39%)	∼0.03	12410
Ir[T*p*CF_3_PC]4pa_2_	836^b^	4.8^b^	∼0.02	818^d^	10.7^d^	∼0.04	12630

^a^In toluene for all complexes except Ir[T*p*CF_3_PC]4pa_2_.

^b^In ethanol.

^c^In toluene/tetrahydrofuran (4:6 v/v) for all complexes except Ir[T*p*CF_3_PC]4pa_2_.

^d^In ethanol/methanol (4:1 v/v)

^e^ Estimated from the blue edge of the emission spectrum at 77 K.

### Singlet oxygen sensitization

**Figure 5:**
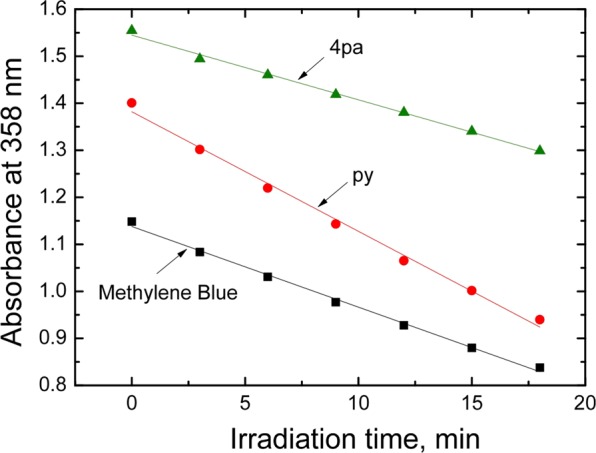
Singlet oxygen-induced degradation of 9,10-dimethylanthracene (c = 0.2  mM) as monitored at 358  nm during photosensitization by two Ir[T*p*CF_3_PC)]L_2_ complexes (L = py and 4pa) and methylene blue in ethanol. Excitation of the sensitizers was performed at 575  nm.

Many metalloporphyrins and related compounds are known to be powerful sensitizers of singlet oxygen owing to efficient intersystem crossing and long triplet state lifetimes^3–9^. The fact that Ir corroles exhibit room temperature phosphorescence indicates that the triplet state is populated to at least some degree. In this study, the Ir-T*p*CF_3_PC complexes with py and 4pa axial ligands were evaluated for their ^1^O_2_ sensitization capabilities. The assay relied on 9,10-dimethylanthracene as a singlet oxygen acceptor^18^. Methylene blue, which exhibits a quantum yield for ^1^O_2_ formation [Φ(^1^O_2_)] of 0.48 and is spectrally compatible with the corroles, was used as the reference^7^. Fig. 5 shows that Ir(III) corroles efficiently sensitize the formation of singlet oxygen. In fact, Φ(^1^O_2_) was found to be 0.71 and 0.38 for L = py and 4pa, respectively. The lower value for 4pa correlates with the lower phosphorescence quantum yield for the same complex (Table 3). These Φ(^1^O_2_) values are several fold higher than those reported for Ir(III) tris(4-cyanophenyl)corrole derivatives, which might reflect more efficient radiationless deactivation of the triplet states of the latter (which exhibit phosphorescence quantum yields of 0.01%)^5^. Efficient sensitization of ^1^O_2_ by Ir(III) complexes is of great interest from the standpoint of photodynamic therapy^19–21^ owing to the co-occurrence of two valuable properties: (i) a long excitation wavelength that enables deeper light penetration and (ii) high flexibility in the choice of axial ligands that should facilitate attachment of tumor markers in the axial positions. Covalent attachment via 4-picolinic acid represents a simple, potential synthetic approach for the latter.

## Conclusion

Although the photophysical studies of six-coordinate Ir(III) corroles have been previously reported, only a handful of complexes have been examined to date, all exhibiting weak NIR phosphorescence at room temperature. Several additional complexes were accordingly synthesized and examined herein, with a view to identifying axial ligands most conducive to NIR phosphorescence. Unfortunately, regardless of the triarylcorrole and the axial ligands (which varied over pyridine, trimethylamine, isoquinoline, 4-dimethylaminopyridine, and 4-picolinic acid), the phosphorescence quantum yield turned out to be very low, estimated at around 0.02–0.04% at ambient temperature, with about a two-fold increase at 77 K. Phosphorescence decay times were found to be around ~5 µs at 295 K and ~10 µs at 77 K. Fortunately, two of the Ir[T*p*CF_3_PC)]L_2_ derivatives were found to efficiently sensitize singlet oxygen formation, with quantum yields Φ(^1^O_2_) = 0.71 and 0.38 for L = py and 4pa, respectively. Iridium corroles thus may hold promise as photosensitizers in photodynamic therapy. The possibility of varying the axial ligand and of attaching tumor-targeting groups at the axial positions makes iridium corroles particularly exciting as drug candidates.

## Experimental Section

### Materials

Unless otherwise mentioned, all chemicals were obtained from Merck. Silica gel 60 (0.04-0.063 mm particle size, 230-400 mesh) was employed for flash chromatography. Silica gel 60 preparative thin-layer chromatographic plates (20 cm ×20 cm, 0.5 mm thick, Merck) and aluminum oxide 60 preparative thin-layer chromatographic plates (20 cm × 20 cm, 1.5 mm thick, Merck) were used for final purification of all complexes. Free-base corroles were prepared according to previously reported procedures^22,23^.

### Instrumental methods

The methods used were essentially the same as in our earlier work^11,13,15,16^. UV − visible spectra were recorded on an HP 8453 spectrophotometer. ^1^H NMR spectra were recorded on a 400 MHz Bruker Avance III HD spectrometer equipped with a 5 mm BB/1H SmartProbe in CDCl_3_ and referenced to residual CHCl_3_ 7.26 ppm, C_6_D_6_ and referenced to residual C_6_H_6_ 7.16 ppm, C_3_D_6_O and referenced to residual C_3_H_6_O 2.05 ppm or CD_3_OD and referenced to residual CH_3_OH 3.31 ppm. High-resolution electrospray-ionization (HR-ESI) mass spectra were recorded from methanolic solution on an LTQ Orbitrap XL spectrometer.

Cyclic voltammetry was performed at 298 K using an EG&G model 263 A potentiostat with a three-electrode system, comprising a glassy carbon working electrode, a platinum wire counter electrode, and a saturated calomel reference electrode (SCE), in CH_2_Cl_2_ (distilled from CaH_2_) as solvent (as in earlier work^11,13,15,16^). The reference electrode was separated from the bulk solution by a fritted-glass bridge filled with the electrolyte solution. The electrolyte solution was purged with argon for several minutes, and electrochemical measurements were conducted under an argon blanket. All potentials are referenced to the SCE.

The phosphorescence of the Ir(III) corroles was studied on a Fluorolog 3 fluorescence spectrometer from Horiba (Japan) equipped with an NIR-sensitive photomultiplier R2658 from Hamamatsu (Japan). The spectra were corrected for the sensitivity of the photomultiplier and smoothing processing (adjusting averaging function) was applied to eliminate noise due to low signals. For measurements at room temperature, dye solutions in a sealable quartz cell (Hellma Analytics, Mülheim, Germany) were deoxygenated by bubbling nitrogen (purity 99.9999%, Linde gas, Austria) for 15 min. Measurements at 77 K were conducted in toluene:tetrahydrofuran (4:6 v/v) or ethanol/methanol (4:1 v/v) frozen glass using accessories for deep-temperature measurements from Horiba. Luminescence decay times were measured on the Fluorolog 3 spectrometer equipped with a DeltaHub module (Horiba Scientific) controlling a SpectraLED-460 light source and using DAS-6 Analysis software for data analysis.

Singlet oxygen generation was studied as previously described^7^. Briefly, a stirred solution containing 9,10-dimethylanthracene (0.2 mM) as a singlet oxygen acceptor and a sensitizer (concentration adjusted to achieve identical absorption for all the sensitizers at λ_ex_) was irradiated with light from the xenon lamp of the Fluorolog spectrometer (λ_ex_ 575 nm). The degradation of the acceptor was assessed via measurement of the UV-vis spectra.

### General procedure for the synthesis of Ir[T*p*XPC]L_2_ (X = OMe, CH_3_, H, CF_3_, L = tma, py, dmap, 4pa, isoq)

The iridium complexes were prepared according to a previously reported procedure^10^ with slight modifications. Bis(1,5-cyclooctadiene)diiridium(I) dichloride (Merck, 2 eq) and potassium carbonate (10 eq.) were dissolved in a solution of the free-base corrole (~0.1 mmol, 1 eq) in anhydrous tetrahydrofuran (150 mL). After degassing with argon for a few minutes, the solution was brought to reflux under argon. After 1.5 h, a reagent corresponding to the axial ligand (15 eq) was added all at once and the solution was left to reach room temperature (1 h). For L = tma, the reagent used was trimethylamine *N*-oxide; in the other cases, the unmodifed ligand was used. The reaction mixture was then rotary-evaporated to dryness. Unless otherwise mentioned, the residue was dissolved in a small amount of dichloromethane and subjected to column chromatography (silica, 1:1 CH_2_Cl_2_:hexanes) followed by preparative thin-layer chromatography (PTLC, silica, 1:1 CH_2_Cl_2_:pentane). Additional details of purification and characterization for each new compound are given below. As in earlier studies of Ir corroles^5,10^, accurate elemental analyses could not be consistently obtained; proof of composition and purity was accordingly obtained via thin-layer chromatography, clean high-resolution mass spectra that matched theoretical simulations, and, in three cases, single-crystal X-ray structure determinations.

### Ir[TPC]tma_2_

PTLC afforded the product as a dichroic purple-green solid. Yield 37.5 mg (30%). UV-vis (CH_2_Cl_2_) λ_max_ (nm) [ϵ x 10^-4^ (M^-1^cm^-1^)]: 384 (3.65, sh), 416 (5.58), 468 (1.59, sh), 596 (2.67). ^1^H NMR (400 MHz, acetone-*d*_6_, δ): 8.88 (d, *J* = 4.1 Hz, 2H, *β*-H), 8.62 (d, *J* = 4.7 Hz, 2H, *β*-H), 8.39 (d, *J* = 4.8 Hz, 2H, *β*-H), 8.24 – 8.19 (m, 4H, 5,15 *o*-Ph), 8.15 – 8.11 (m, 2H, 10 *o*-Ph), 8.01 (s, 2H, *β*-H), 7.78 (dt, *J* = 14.9, 7.5 Hz, 6H, 5,10,15 *m*-Ph), 7.69 – 7.63 (m, 3H, 5,10,15 *p*-Ph), -2.86 (s, 18H, tma-CH_3_). MS (ESI): M^+^ = 834.3017 (expt), 834.3019 (calcd for IrC_43_H_41_N_6_).

### Ir[TPC]py_2_

PTLC afforded the product as a dichroic purple-green solid. Yield 83.4 mg (63.6%). UV-vis (CH_2_Cl_2_) λ_max_ (nm) [ϵ x 10^-4^ (M^-1^cm^-1^)]: 414 (5.30), 459 (2.66, sh), 562 (0.86, sh), 602 (3.37). ^1^H NMR (400 MHz, acetone-*d*_6_, δ): 8.82 – 8.78 (m, 2H, *β*-H), 8.62 (dd, *J* = 4.8, 1.1 Hz, 2H, *β*-H), 8.38 (d, *J* = 4.7 Hz, 2H, *β*-H), 8.28 – 8.22 (m, 4H, 5,15 *o*-Ph), 8.17 (d, *J* = 4.1 Hz, 2H, *β*-H), 8.05 – 8.00 (m, 2H, 10 *o*-Ph), 7.77 (dd, *J* = 8.3, 6.8 Hz, 4H, 5,15 *m*-Ph), 7.70 – 7.59 (m, 5H, 5,15 *p*-Ph and 10 *p*,*m*-Ph), 6.30 (tt, *J* = 7.6, 1.5 Hz, 2H, *p*-py), 5.42 – 5.33 (m, 4H, *m*-py), 1.85 (dt, *J* = 5.4, 1.5 Hz, 4 H, *o*-py). MS (ESI): M^+^ = 874.2403 (expt), 874.2393 (calcd for IrC_47_H_33_N_6_).

### Ir[T*p*MePC]tma_2_

PTLC with 1:2 CH_2_Cl_2_:pentane afforded the product as a bright green solid. Yield 75.7 mg (49.6%). UV-vis (CH_2_Cl_2_) λ_max_ (nm) [ϵ x 10^-4^ (M^-1^cm^-1^)]: 384 (3.28, sh), 417 (4.88), 468 (1.54, sh), 598 (2.36). ^1^H NMR (400 MHz, chloroform-*d*, δ): 8.88 – 8.82 (m, 2H, *β*-H), 8.66 (d, *J* = 4.7 Hz, 2H, *β*-H), 8.39 (d, *J* = 4.6 Hz, 2H, *β*-H), 8.03 (dd, *J* = 47.5, 7.5 Hz, 8H 5,10,15 *o*-Ph + *β*-H, 8H), 7.57 (dd, *J* = 14.0, 7.6 Hz, 6H, 5,10,15 *m*-Ph), 1.28 (s, 9H, Ph *p*-CH_3_), -2.88 (s, 18H, tma-CH_3_). MS (ESI): M^+^ = 876.3488 (expt), 876.3489 (calcd for IrC_46_H_47_N_6_).

### Ir[T*p*MePC]py_2_

PTLC afforded the product as a dichroic purple-green solid. Yield 36 mg (30.1%). UV-vis (CH_2_Cl_2_) λ_max_ (nm) [ϵ × 10^-4^ (M^-1^cm^-1^)]: 417 (6.40), 460 (2.75, sh), 562 (0.98, sh), 603 (3.49). ^1^H NMR (400 MHz, benzene-*d*_6_, δ): 9.14 (dd, *J* = 4.5, 3.0 Hz, 4H, *β*-H), 8.97 (d, *J* = 4.8 Hz, 2H, *β*-H), 8.80 (d, *J* = 4.2 Hz, 2H, *β*-H), 8.46 – 8.41 (m, 4H, 5,15 *o*-Ph), 8.30 (d, *J* = 7.9 Hz, 2H, 10 *o*-Ph), 7.42 (d, *J* = 7.7 Hz, 4H, 5,15 *m*-Ph), 7.37 (d, *J* = 8.0 Hz, 2H, 10 *m*-Ph), 4.79 (t, *J* = 7.6 Hz, 2H, *p*-py), 4.17 – 4.11 (m, 4H, *m*-py), 2.42 (d, *J* = 4.2 Hz, 9H, Ph -*p*-CH_3_), 2.06 (dt, *J* = 5.3, 1.5 Hz, 4H, *o*-py). MS (ESI): M^+^ = 916.2868 (expt), 916.2863 (calcd for IrC_50_H_39_N_6_).

### Ir[T*p*OMePC]tma_2_

During initial column chromatography on silica, the eluent was gradually changed from 1:1 CH_2_Cl_2_:hexanes to CH_2_Cl_2_, and finally to 9:1 CH_2_Cl_2_:EtOAc. PTLC with 4:1 EtOAc:CH_2_Cl_2_ then afforded the product as a bright green solid. Yield 35.2 mg (29.2%). UV-vis (CH_2_Cl_2_) λ_max_ (nm) [ϵ x 10^-4^ (M^-1^cm^-1^)]: 384 (0.74, sh), 417 (1.05), 468 (0.36, sh), 600 (0.48). ^1^H NMR (400 MHz, chloroform-*d*, δ): 8.89 (d, *J* = 3.8 Hz, 2H, *β*-H), 8.68 (d, *J* = 4.7 Hz, 2H, *β*-H), 8.32 (d, *J* = 4.6 Hz, 2H, *β*-H), 8.06 (d, *J* = 8.0 Hz, 4H, 5,15 *o*-Ph), 7.92 (d, *J* = 7.9 Hz, 2H, 10 *o*-Ph), 7.83 (s, 2H, *β*-H), 7.33 (dd, *J* = 11.7, 8.2 Hz, 6H, 5,10,15 *m*-Ph), 4.09 (d, *J* = 3.6 Hz, 9H, Ph *p*-OCH_3_), -2.86 (s, 18H, tma-CH_3_). MS (ESI): M^+^ = 924.3336 (expt), 924.3336 (calcd for IrC_46_H_47_O_3_N_6_).

### Ir[T*p*OMePC]py_2_

PTLC afforded the product as a bright green solid. Yield 31.2 mg (24.5%). UV-vis (CH_2_Cl_2_) λ_max_ (nm) [ϵ x 10^-4^ (M^-1^cm^-1^)]: 416 (3.98), 458 (2.14, sh), 563 (0.77, sh), 605 (2.36). ^1^H NMR (400 MHz, benzene-*d*_6_, δ): 9.19 – 9.15 (m, 4H, 2 *β*-H), 8.98 (d, *J* = 4.9 Hz, 2H, *β*-H), 8.76 (s, 2H, *β*-H), 8.42 (d, *J* = 8.0 Hz, 4H, 5,15 *o*-Ph), 8.08 (d, *J* = 8.5 Hz, 2H, 10 *o*-Ph), 7.20 (d, *J* = 8.8 Hz, 4H, 5,15 *m*-Ph), 7.11 – 7.07 (m, 2H, 10 *m*-Ph), 4.81 (ddd, *J* = 7.7, 6.2, 1.5 Hz, 2H, *p*-py), 4.20 – 4.16 (m, 4H, *m*-py), 3.55 (d, *J* = 2.0 Hz, 9H, Ph *p*-OCH_3_), 2.11 – 2.07 (m, 4H, *o*-py). MS (ESI): M^+^ = 964.2738 (expt), 964.2710 (calcd for IrC_50_H_39_O_3_N_6_).

### Ir[T*p*CF_3_PC]tma_2_

PTLC with 1:3 CH_2_Cl_2_:pentane afforded the product as a dark green solid. Yield 14.7 mg (11.8%). UV-vis (CH_2_Cl_2_) λ_max_ (nm) [ϵ x 10^-4^ (M^-1^cm^-1^)]: 418 (2.13), 595 (0.68). ^1^H NMR (400 MHz, chloroform-*d*, δ): 8.89 (d, *J* = 4.2 Hz, 2H, *β*-H), 8.64 (d, *J* = 4.8 Hz, 2H, *β*-H), 8.48 (d, *J* = 4.8 Hz, 2H, *β*-H), 8.38 (d, *J* = 7.8 Hz, 4H, 5,15 *o*-Ph), 8.29 (d, *J* = 7.8 Hz, 2H, 10 *o*-Ph), 8.20 (d, *J* = 4.2 Hz, 2H, *β*-H), 8.01 (d, *J* = 7.9 Hz, 4H, 5,15 *m*-Ph), 7.98 (d, *J* = 7.9 Hz, 2H, 10 *o*-Ph), -2.93 (s, 18H, tma-CH_3_). MS (ESI): M^+^ = 1038.2654 (expt), 1038.2641 (calcd for IrC_46_H_38_F_9_N_6_).

### Ir[T*p*CF_3_PC]py_2_

PTLC with 1:2 CH_2_Cl_2_:pentane afforded the product as a dark green solid. Yield 16.3 mg (13.7%). UV-vis (CH_2_Cl_2_) λ_max_ (nm) [ϵ x 10^-4^ (M^-1^cm^-1^)]: 416 (3.86), 602 (1.48). ^1^H NMR (400 MHz, benzene-*d*_6_, δ): 9.12 (d, *J* = 4.3 Hz, 2H, *β*-H), 8.90 (d, *J* = 4.8 Hz, 2H, *β*-H), 8.75 (d, *J* = 4.8 Hz, 2H, *β*-H), 8.54 (d, *J* = 4.3 Hz, 2H, *β*-H), 8.30 (d, *J* = 8.0 Hz, 4H, 5,15 *o*-Ph), 8.23 (d, *J* = 7.9 Hz, 2H, 10 *o*-Ph), 7.76 (dd, *J* = 8.1, 6.6 Hz, 6H, 5,10,15 *m*-Ph), 4.84 (tt, *J* = 7.7, 1.5 Hz, 2H, *p*-py), 4.23 – 4.17 (m, 4H, *m*-py), 1.86 (dt, *J* = 5.6, 1.5 Hz, 4H, *o*-py). MS (ESI): M^+^ = 1078.2032 (expt), 1078.2015 (calcd for IrC_50_H_30_F_9_N_6_).

### Ir[T*p*CF_3_PC]dmap_2_

Initial column chromatography was carried out on neutral alumina with 1:1 CH_2_Cl_2_:hexanes as eluent. PTLC on alumina with the same eluent afforded the product as a green solid. Yield 31.7 mg (18.1%). UV-vis (CH_2_Cl_2_) λ_max_ (nm) [ϵ x 10^-4^ (M^-1^cm^-1^)]: 418 (1.79), 606 (0.48). ^1^H NMR (400 MHz, benzene-*d*_6_, δ): 9.20 (d, *J* = 4.2 Hz, 2H, *β*-H), 8.99 (d, *J* = 4.8 Hz, 2H, *β*-H), 8.86 (d, *J* = 4.8 Hz, 2H, *β*-H), 8.60 (d, *J* = 4.2 Hz, 2 H, *β*-H), 8.45 (d, *J* = 7.9 Hz, 4 H, 5,15 *o*-Ph), 8.40 (d, *J* = 7.9 Hz, 2H, 10 *o*-Ph), 7.77 (dd, *J* = 8.3, 2.1 Hz, 6H, 5,10,15 *m*-Ph), 3.57 – 3.52 (m, 4H, *m*-py), 1.76 – 1.69 (m, 4 H, *o*-Ph), 1.07 (s, 6 H, *N*-CH_3_), 1.00 (s, 6H, *N*-CH_3_). MS (ESI): M^+^ = 1164.2855 (expt), 1164.2860 (calcd for IrC_54_H_40_F_9_N_8_).

### Ir[T*p*CF_3_PC](4pa)_2_

Initial column chromatography was carried out on neutral alumina with 1:1 CH_2_Cl_2_:EtOAc. PTLC on alumina with the same eluent afforded the product as a green solid. Yield 29.6 mg (40.1%). UV-vis (CH_2_Cl_2_) λ_max_ (nm) [ϵ x 10^-4^ (M^-1^cm^-1^)]: 414 (1.71), 602 (0.94). ^1^H NMR (400 MHz, methanol-*d*_4_, δ): 8.88 (d, *J* = 4.2 Hz, 2 H, *β*-H), 8.61 (d, *J* = 4.8 Hz, 2 H, *β*-H), 8.43 – 8.41 (m, 6 H, *β*-H + 5,15 *o*-Ph), 8.28 (d, *J* = 8.0 Hz, 2 H, 10 *o*-Ph), 8.24 (d, *J* = 4.3 Hz, 2 H, *β*-H), 8.08 – 8.03 (m, 4 H, 5,15 *m*-Ph), 7.99 (d, *J* = 7.9 Hz, 2 H, 10 *m*-Ph), 5.61 (d, *J* = 7.1 Hz, 4 H, *m*-py), 1.75 (d, *J* = 7.0 Hz, 4 H, *o*-py). MS (ESI): M^–^ = 1165.1729 (expt), 1165.1745 (calcd for IrC_52_H_29_F_9_N_6_O_4_).

### Ir[T*p*CF3PC]isoq_2_

Initial column chromatography was carried out on silica with 1:3 CH_2_Cl_2_:hexanes as eluent. PTLC on silica with the same eluent afforded the product as a green solid. Yield 12.1 mg (7.9%). UV-vis (CH_2_Cl_2_) λ_max_ (nm) [ϵ x 10^-4^ (M^-1^cm^-1^)]: 417 (1.82), 603 (1.02). ^1^H NMR (400 MHz, chloroform-*d*, δ): 8.88 (d, *J* = 4.2 Hz, 2 H, *β*-H), 8.69 (d, *J* = 4.8 Hz, 2 H, *β*-H), 8.46 (d, *J* = 4.7 Hz, 2 H, *β*-H), 8.43 (d, *J* = 7.9 Hz, 4 H, 5,15 *o*-Ph), 8.29 (s, 2 H, *β*-H), 8.24 (d, *J* = 7.9 Hz, 2 H, 10 *o*-Ph), 8.05 – 7.99 (m, 4 H, 5,15 *m*-Ph), 7.93 (d, *J* = 7.9 Hz, 2 H, 10 *m*-Ph), 7.07 (ddd, *J* = 8.2, 6.9, 1.2 Hz, 2 H, isoquinoline C7), 6.93 (ddd, *J* = 8.2, 6.9, 1.1 Hz, 2 H, isoquinoline C6), 6.76 – 6.72 (m, 2H, isoquinoline C8), 6.40 (d, *J* = 8.4 Hz, 2 H, isoquinoline C5), 5.58 – 5.54 (m, 2 H, isoquinoline C4), 2.36 (s, 2 H, isoquinoline C1), 1.68 (d, *J* = 6.9 Hz, 2 H, isoquinoline C3). MS (ESI): M^–^ = 1178.2351 (expt), 1178.2329 (calcd for IrC_58_H_34_F_9_N_6_).

### X-ray structure determinations

X-ray data for Ir[TPC]tma_2_, Ir[T*p*MePC]tma_2_, and Ir[T*p*CF_3_PC]py_2_ were collected on beamline 11.3.1 at the Advanced Light Source, Lawrence Berkeley National Laboratory. Samples were mounted on MiTeGen kapton loops and placed in a 100(2)-K (for Ir[TPC]tma_2_ and Ir[T*p*CF_3_PC]py_2_) or 150(2)-K (for Ir[T*p*CF_3_PC]py_2_) nitrogen cold stream provided by an Oxford Cryostream 800 Plus low-temperature apparatus on the goniometer head of a Bruker D8 diffractometer equipped with a PHOTON100 CMOS detector operating in shutterless mode. Diffraction data were collected using synchrotron radiation monochromated using silicon(111) to a wavelength of 0.7293(1)Å (for Ir[T*p*MePC]tma_2_) or 0.7749(1)Å (for Ir[TPC]tma_2_ and Ir[T*p*CF_3_PC]py_2_). An approximate full-sphere of data was collected using a combination of *φ* and *ω* scans with scan speeds of one second per degree for the φ scans and one and three seconds per degree for the ω scans at 2*θ* = 0° and −45°, respectively. The structures were solved by intrinsic phasing (SHELXT^24^) and refined by full-matrix least-squares on *F*^2^ (SHELXL-2014^25^). All non-hydrogen atoms were refined anisotropically. Hydrogen atoms were geometrically calculated and refined as riding atoms. Additional crystallographic information has been summarized in Table 2.

## Supplementary information

Supplementary Information.
