# Supplementary material for: Iridium Corroles Exhibit Weak Near-Infrared Phosphorescence but Efficiently Sensitize Singlet Oxygen Formation
Source: Sci Rep. 2020 May 5;10:7551. doi: 10.1038/s41598-020-64389-3 (PMC7200656; doi:10.1038/s41598-020-64389-3)
Supplement: Supplementary file 1 — Supplementary Information. [file 41598_2020_64389_MOESM1_ESM.pdf]

Supporting Information

Iridium Corroles Exhibit Weak Near-Infrared Phosphorescence but Efficiently Sensitize Singlet Oxygen Formation

Ivar K. Thomassen,[a] Laura J. McCormick-McPherson,[b] Sergey M. Borisov[c] and Abhik Ghosh*,[a]

[a]Department of Chemistry, UiT – The Arctic University of Norway, N-9037 Tromsø, Norway; Email: abhik.ghosh@uit.no.

[b]Advanced Light Source, Lawrence Berkeley National Laboratory, Berkeley, California 94720-8229, United States

[c]Institute of Analytical Chemistry and Food Chemistry, Graz University of Technology, Stremayrgasse 9, 8010 Graz, Austria; Email: sergey.borisov@tugraz.at

Table of Contents

A. UV-vis spectra S2

B. Mass spectra S7

C. ^{1}H NMR spectra S18

A. UV-vis spectra

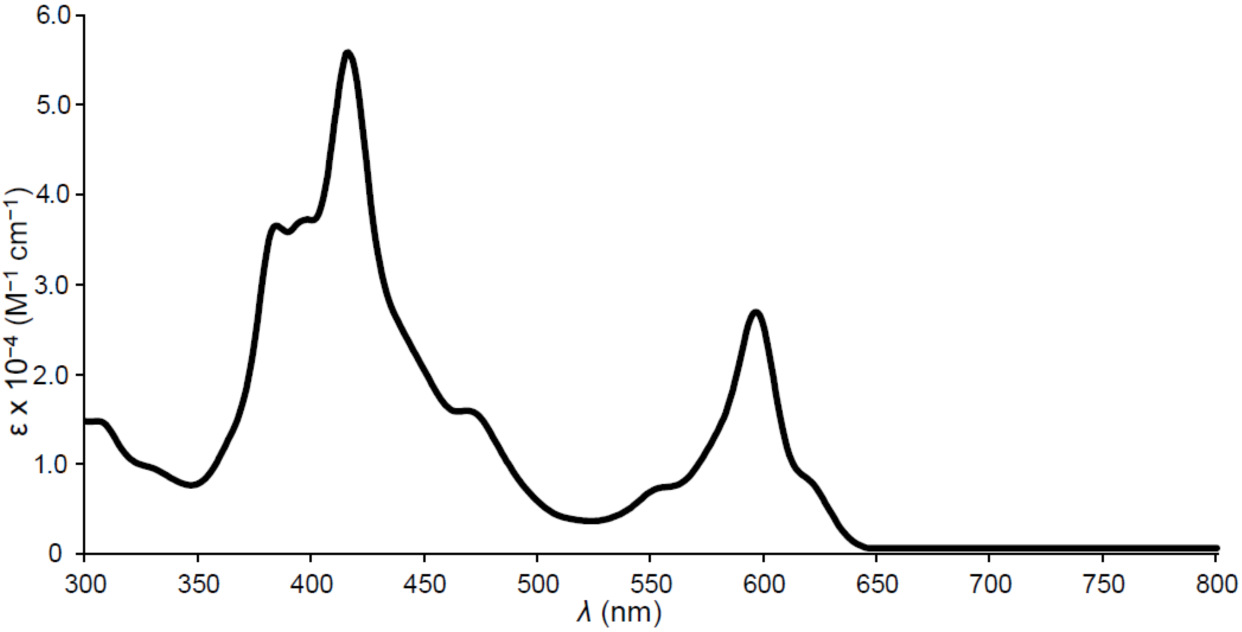

Figure 1. UV-vis spectrum of Ir[TPC]tma$_2$.

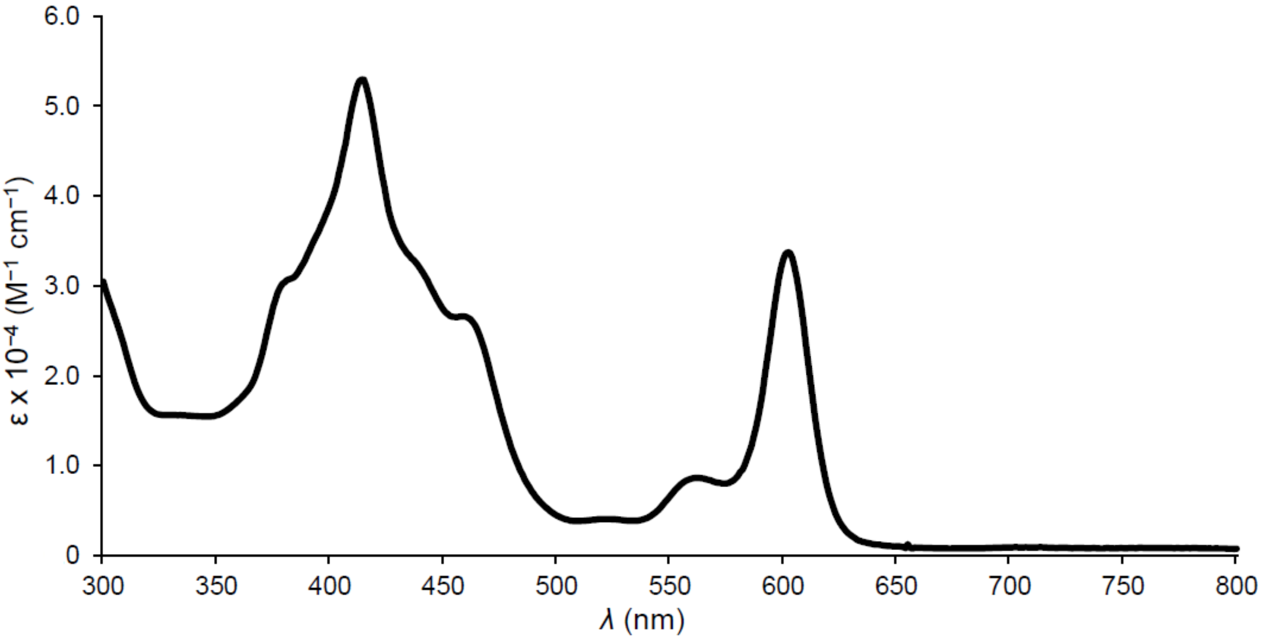

Figure 2. UV-vis spectrum of Ir[TPC]py$_2$.

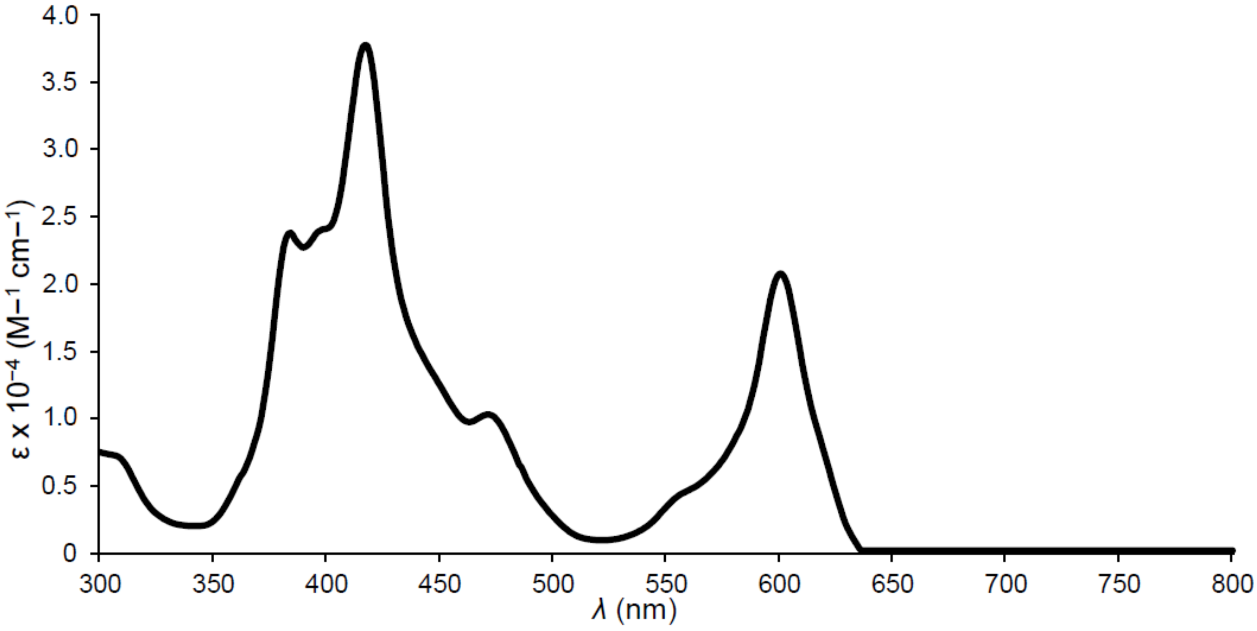

Figure 3. UV-vis spectrum of Ir[T*p*OMePC]tma$_2$.

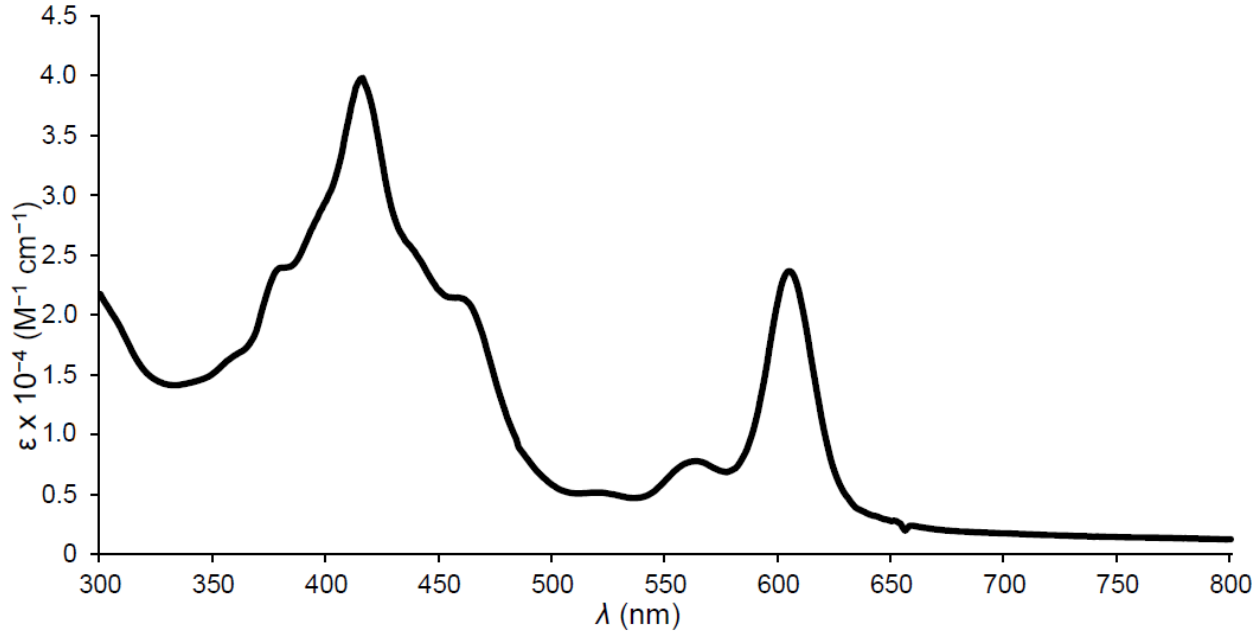

Figure 4. UV-vis spectrum of Ir[T*p*OMePC]py$_2$.

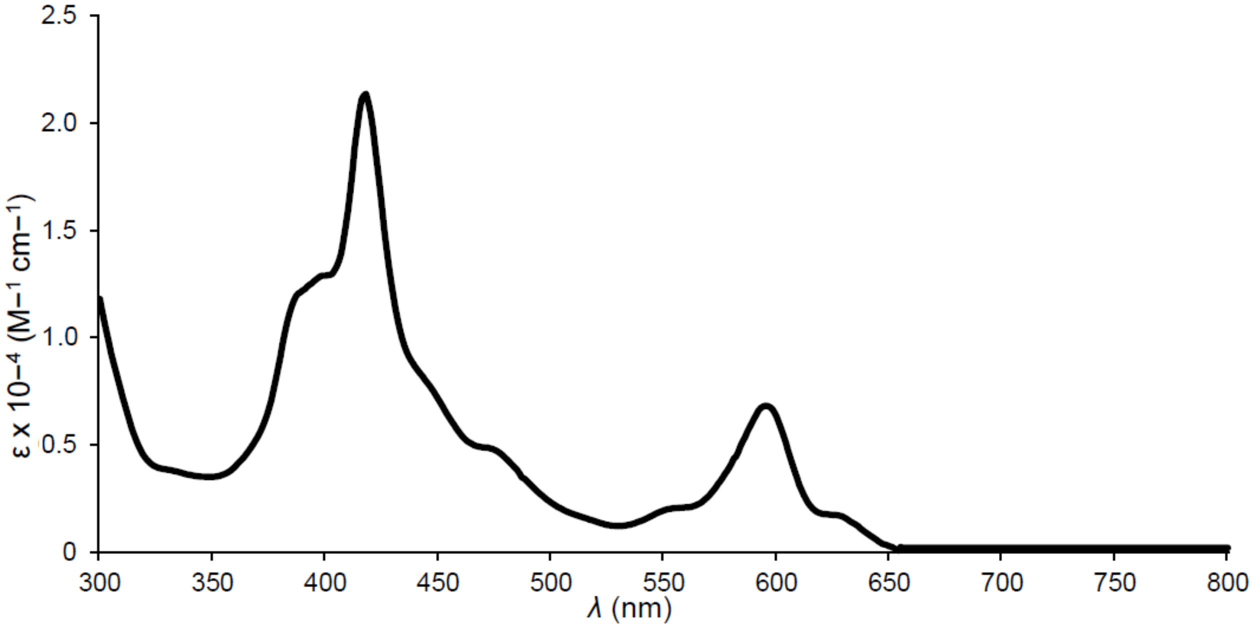

Figure 5. UV-vis spectrum of Ir[T*p*CF_3PC]tma_2.

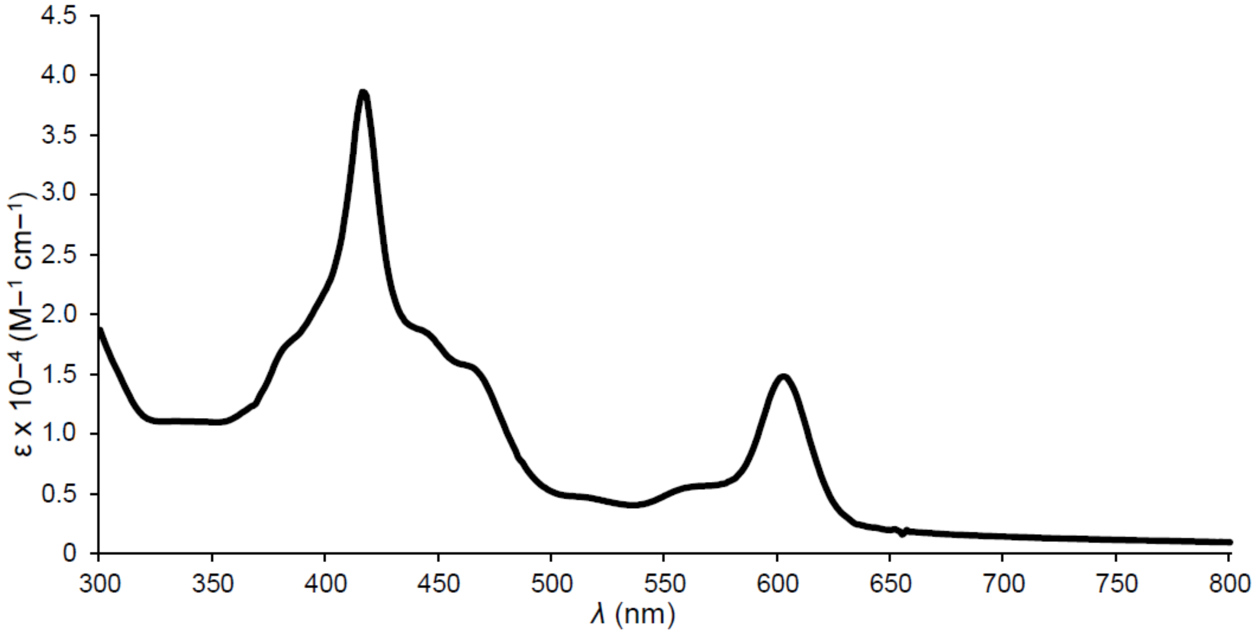

Figure 6. UV-vis spectrum of Ir[T*p*CF_3PC]py_2.

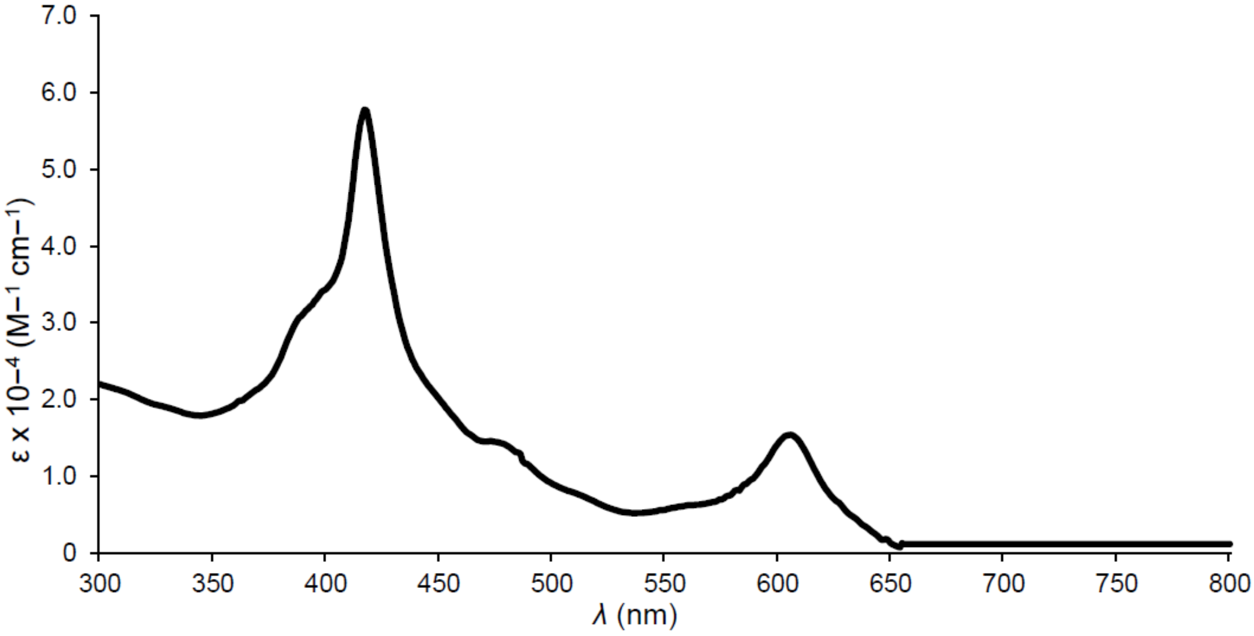

Figure 7. UV-vis spectrum of Ir[T*p*CF_3PC]dmap$_2$.

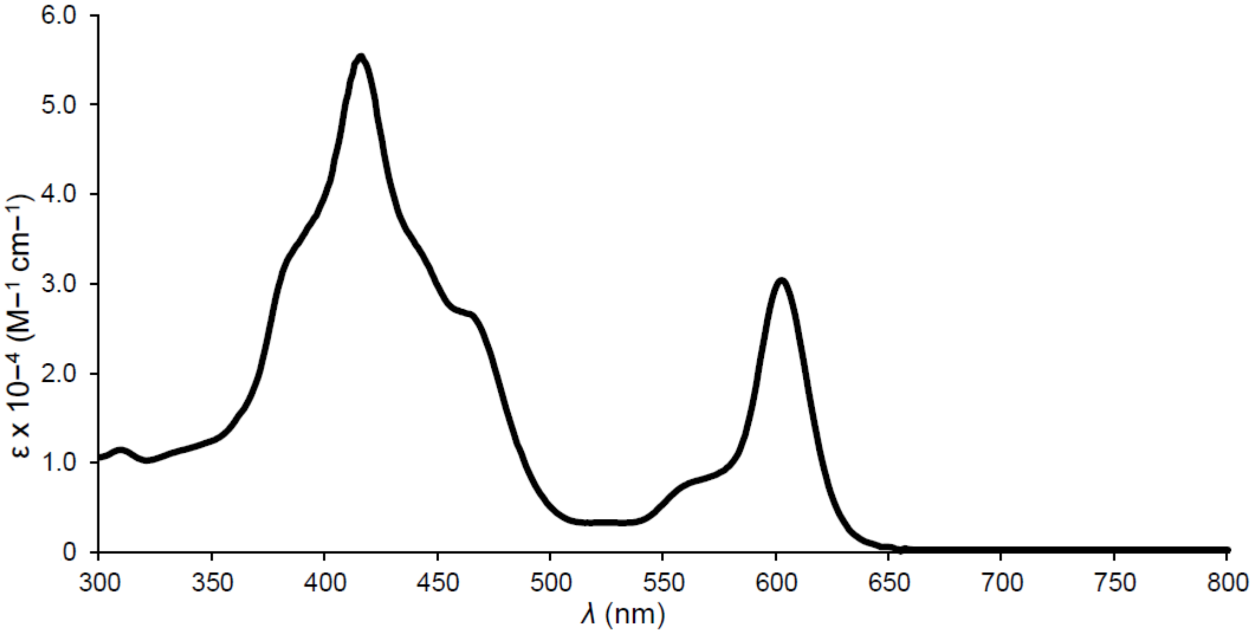

Figure 8. UV-vis spectrum of Ir[T*p*CF_3PC]4pa$_2$.

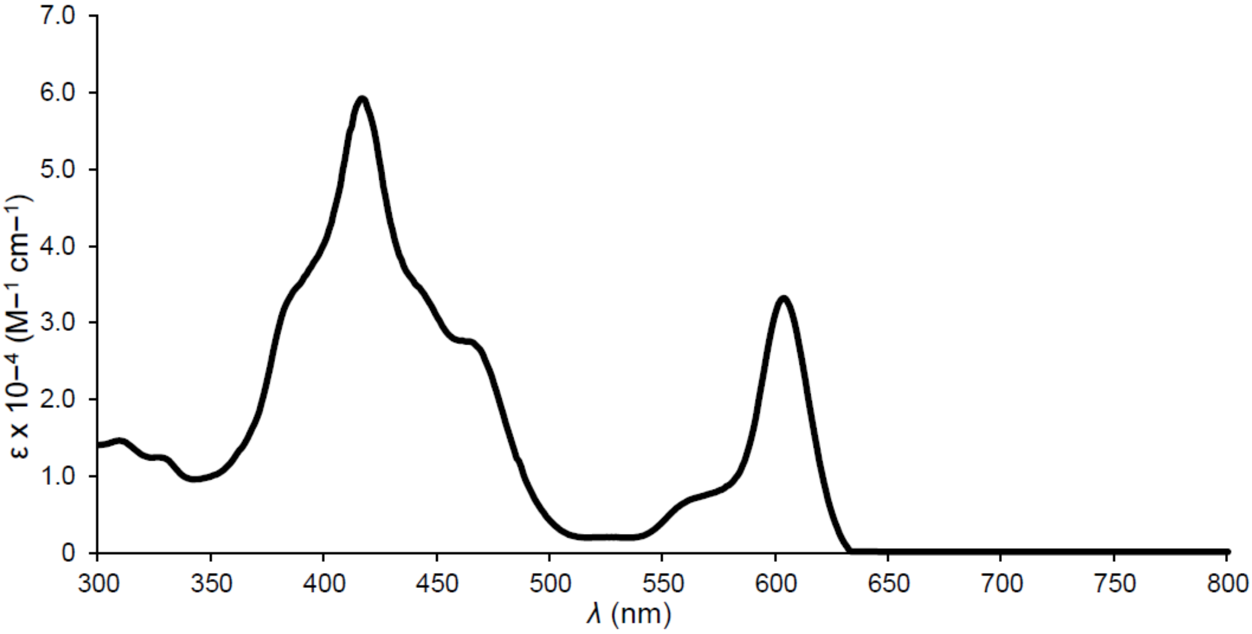

Figure 9. UV-vis spectrum of Ir[T*p*CF_3PC]isoq$_2$.

B. Mass spectra

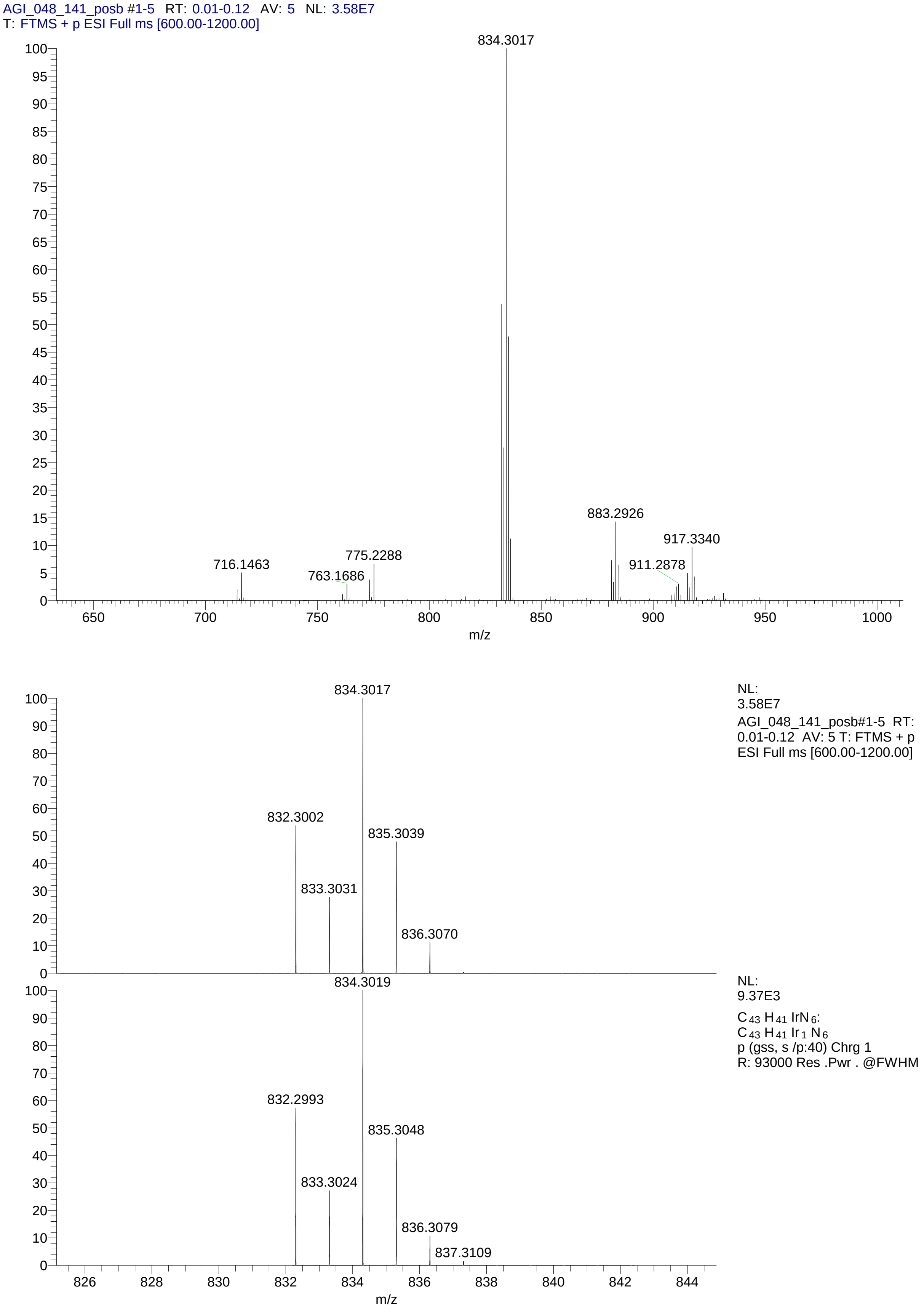

Figure 10. Electrospray ionization mass spectrum of Ir[TPC]tma$_2$.

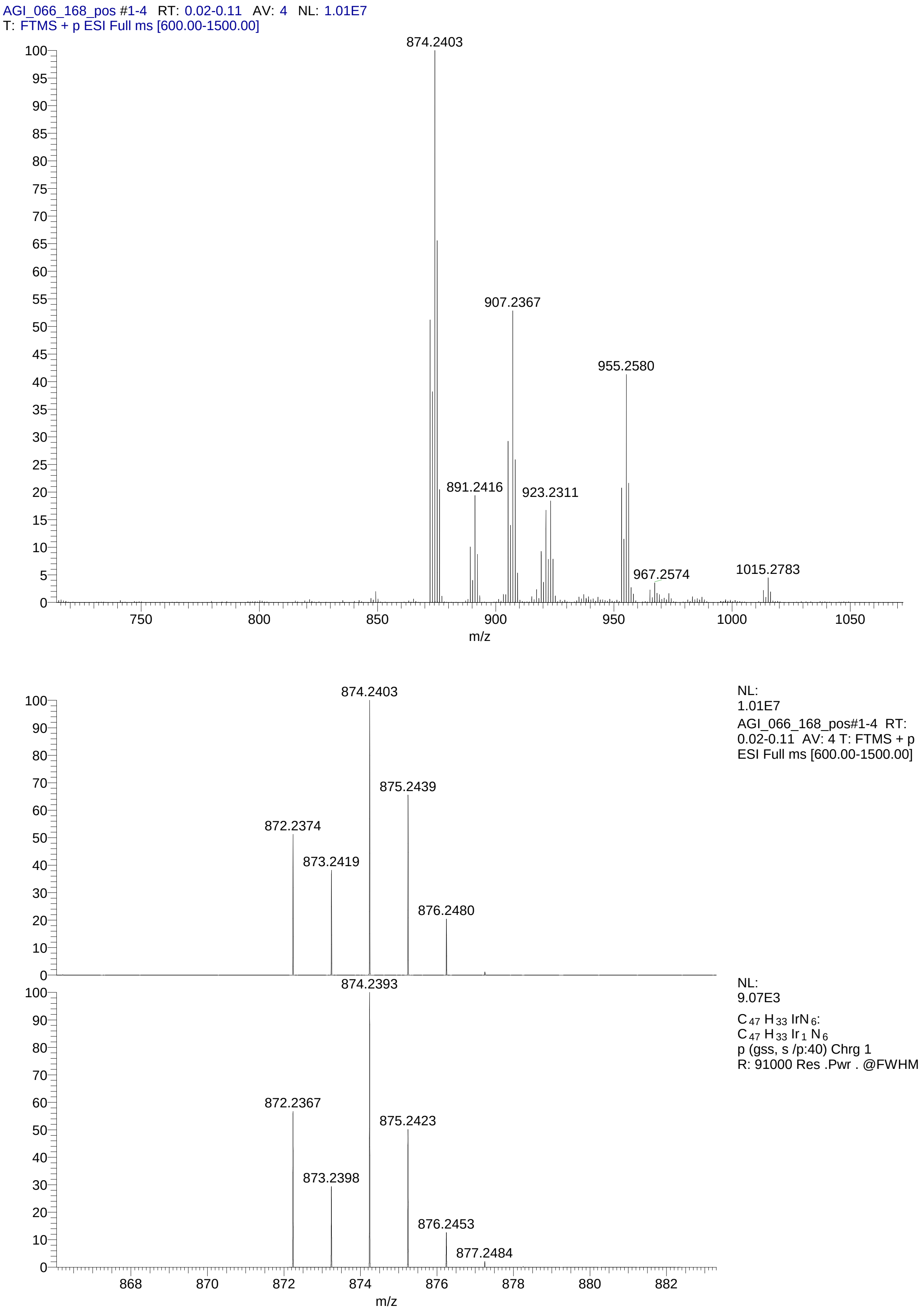

Figure 11. Electrospray ionization mass spectrum of Ir[TPC]py$_2$.

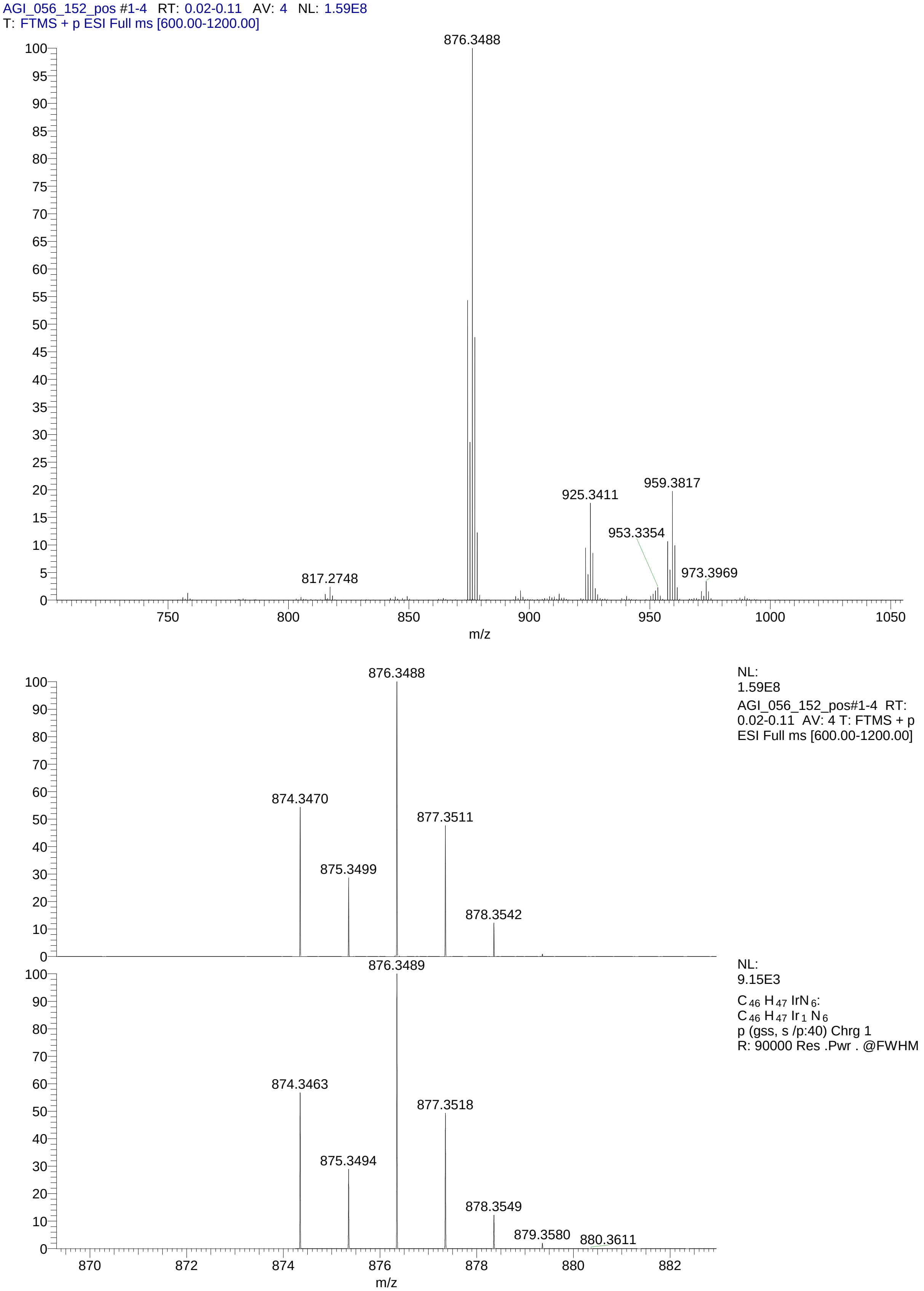

Figure 12. Electrospray ionization mass spectrum of Ir[T*p*MePC]tma$_2$.

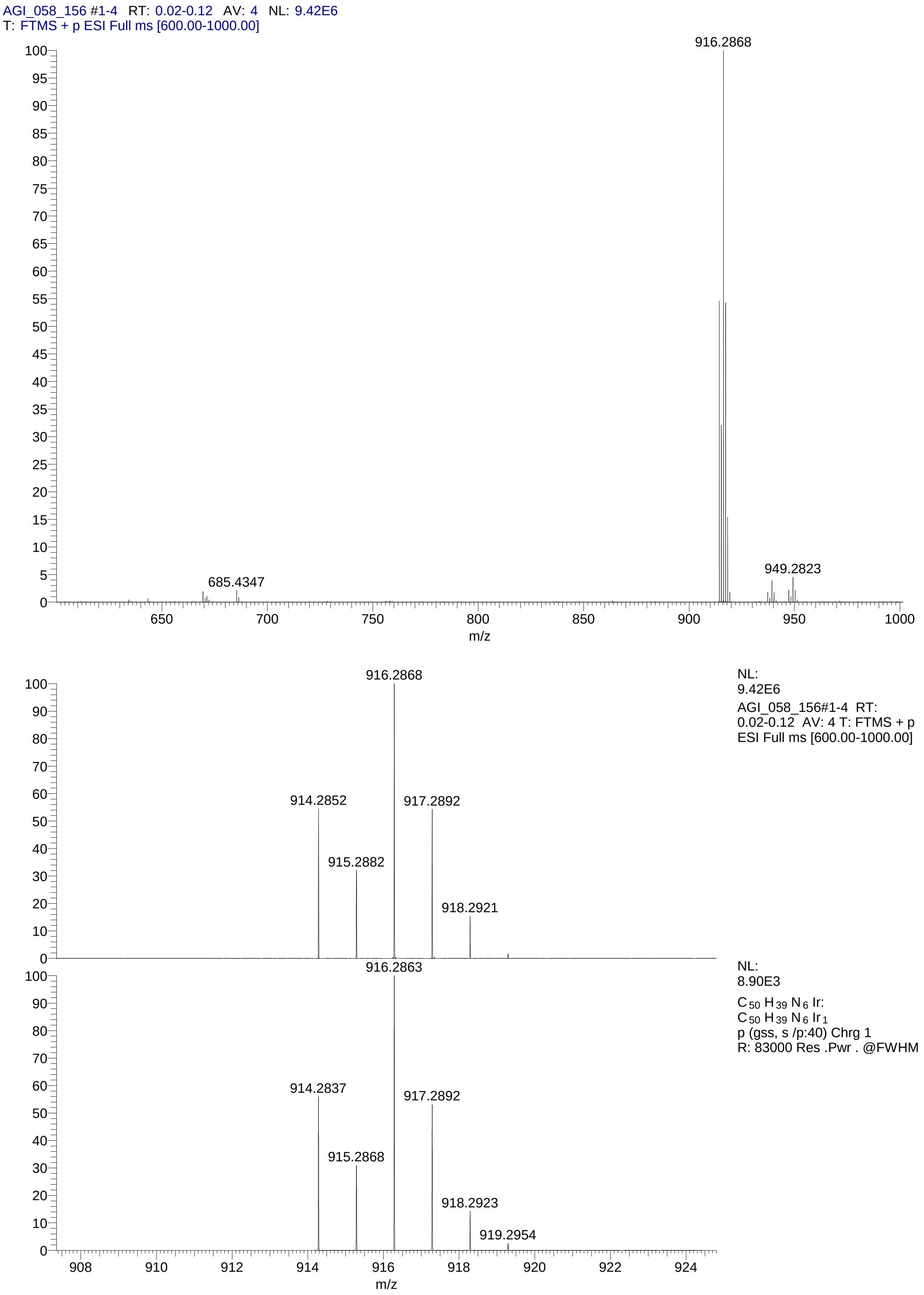

Figure 13. Electrospray ionization mass spectrum of Ir[T*p*MePC]py$_2$.

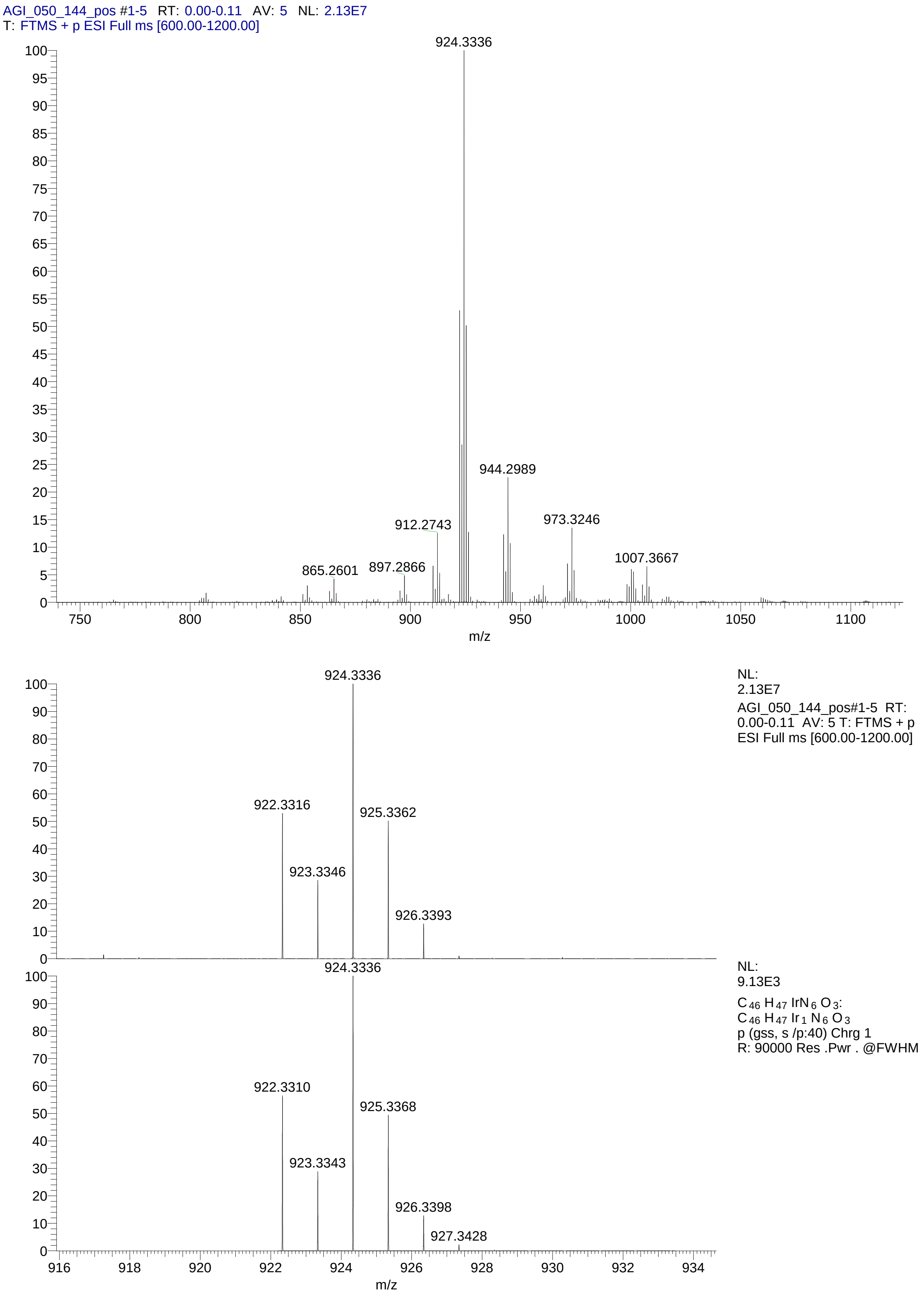

Figure 14. Electrospray ionization mass spectrum of Ir[T*p*OMePC]tma$_2$.

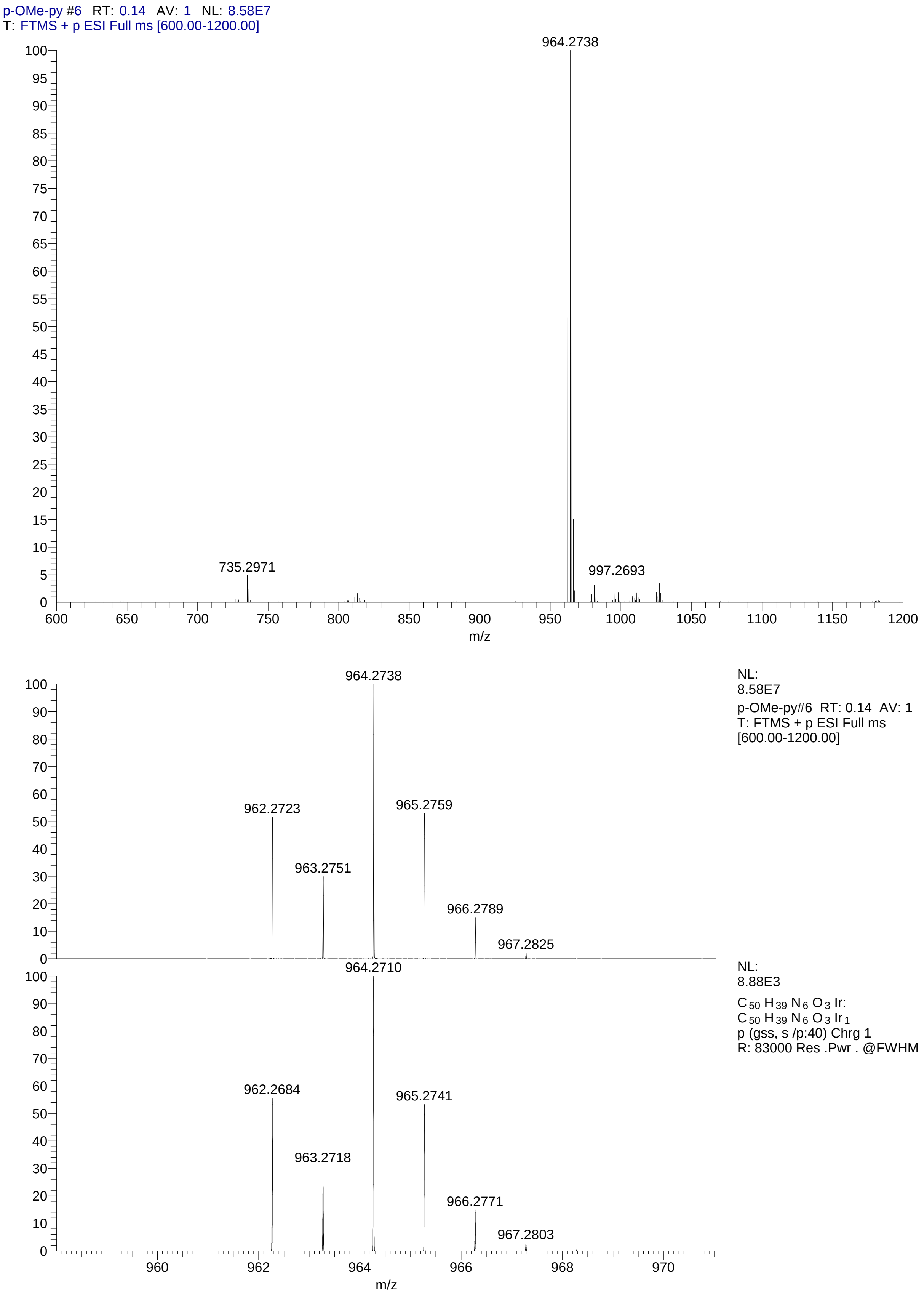

Figure 15. Electrospray ionization mass spectrum of Ir[T*p*OMePC]py$_2$.

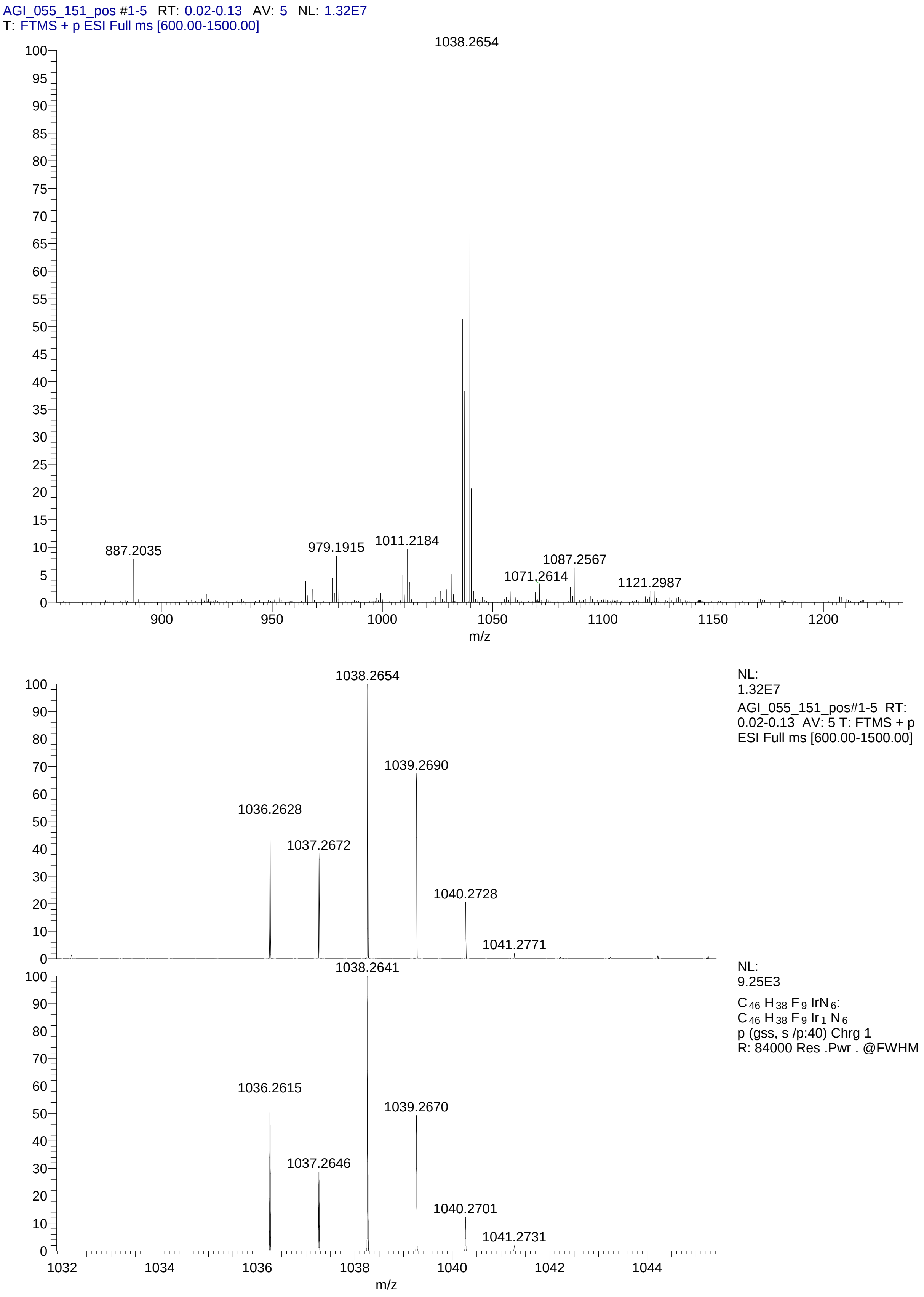

Figure 16. Electrospray ionization mass spectrum of Ir[T*p*CF_3PC]tma_2.

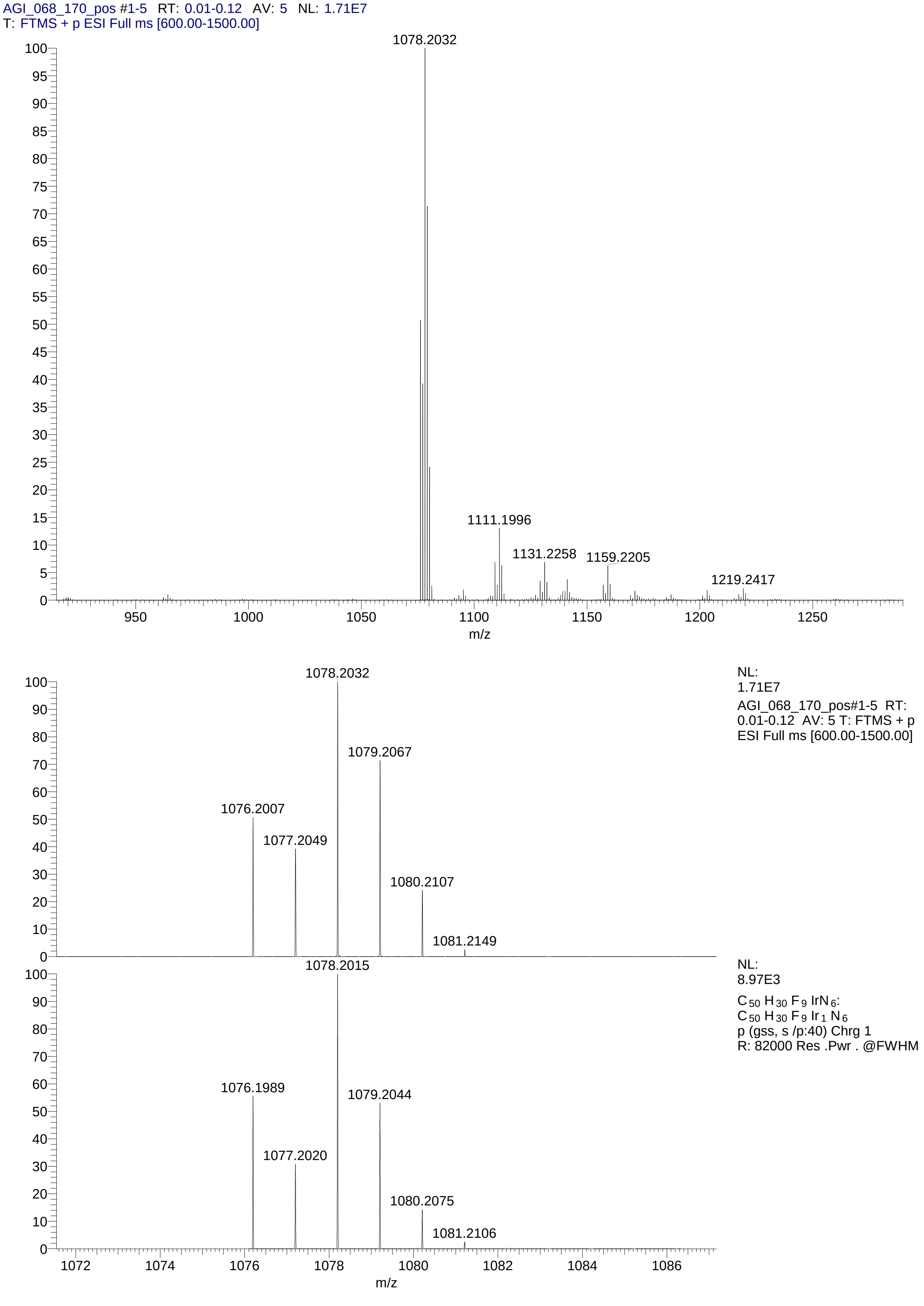

Figure 17. Electrospray ionization mass spectrum of Ir[T*p*CF_3PC]py_2.

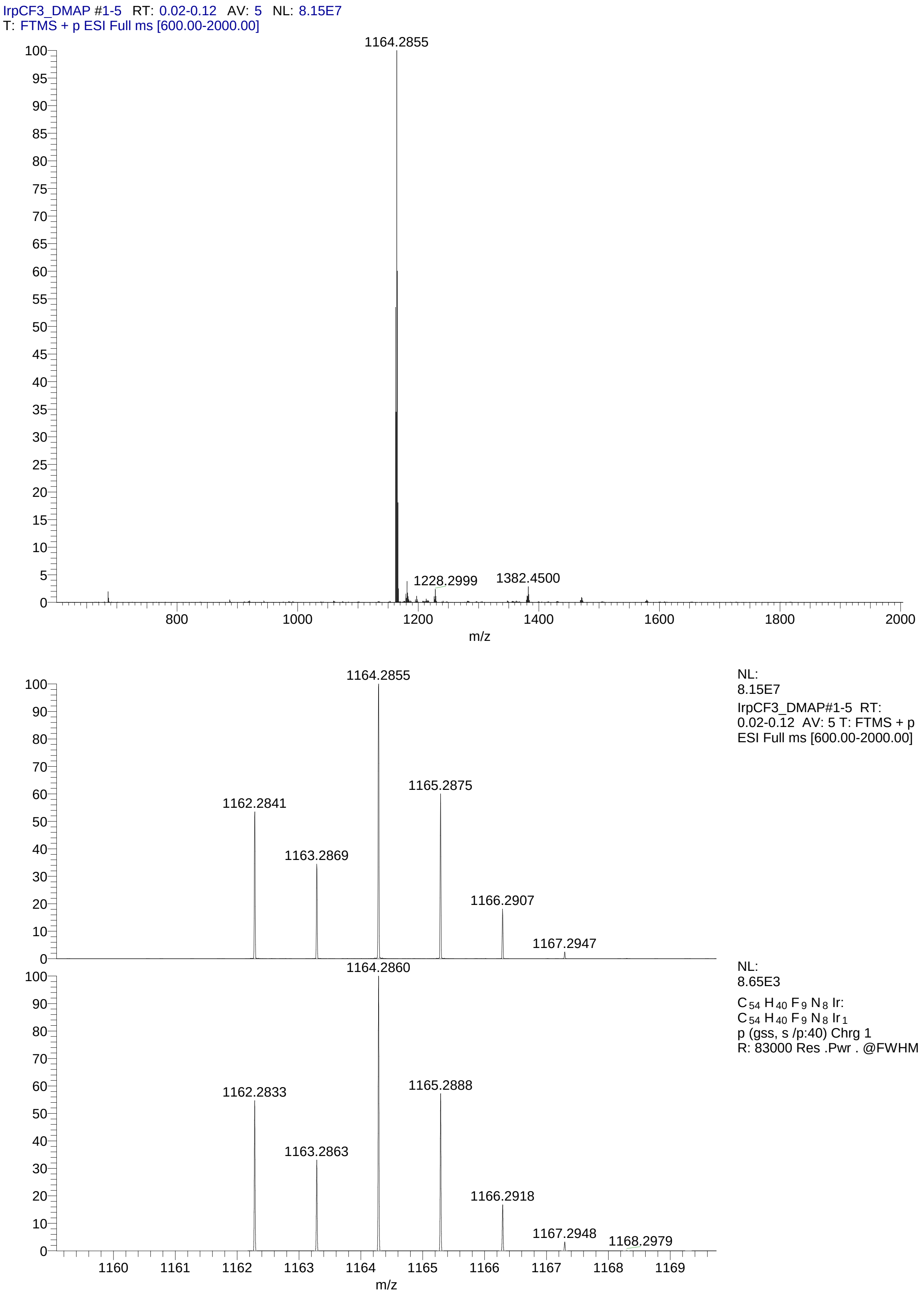

Figure 18. Electrospray ionization mass spectrum of Ir[T*p*CF_3PC]$dmap_2$.

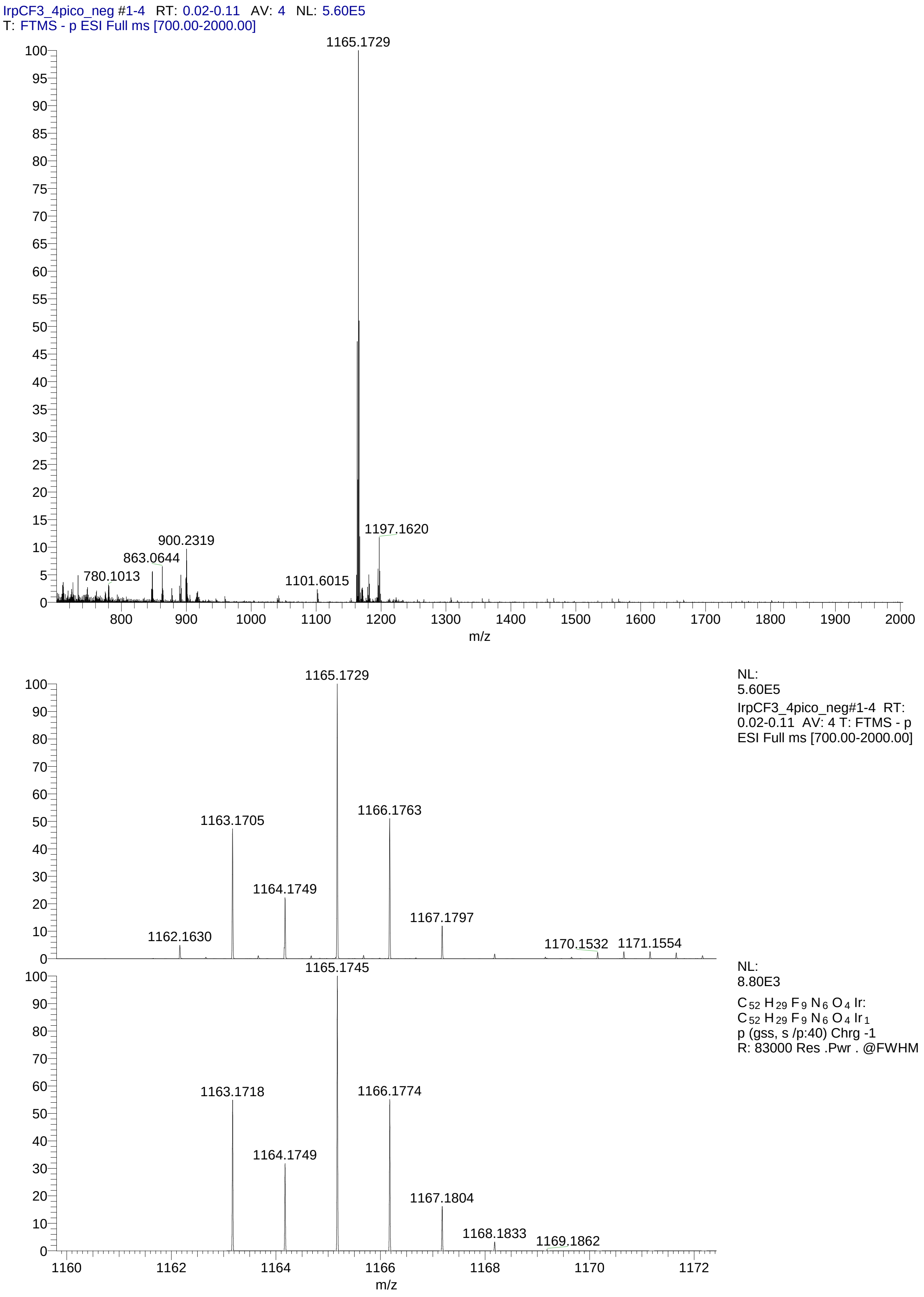

Figure 19. Electrospray ionization mass spectrum of Ir[T*p*CF_3PC]4pa_2.

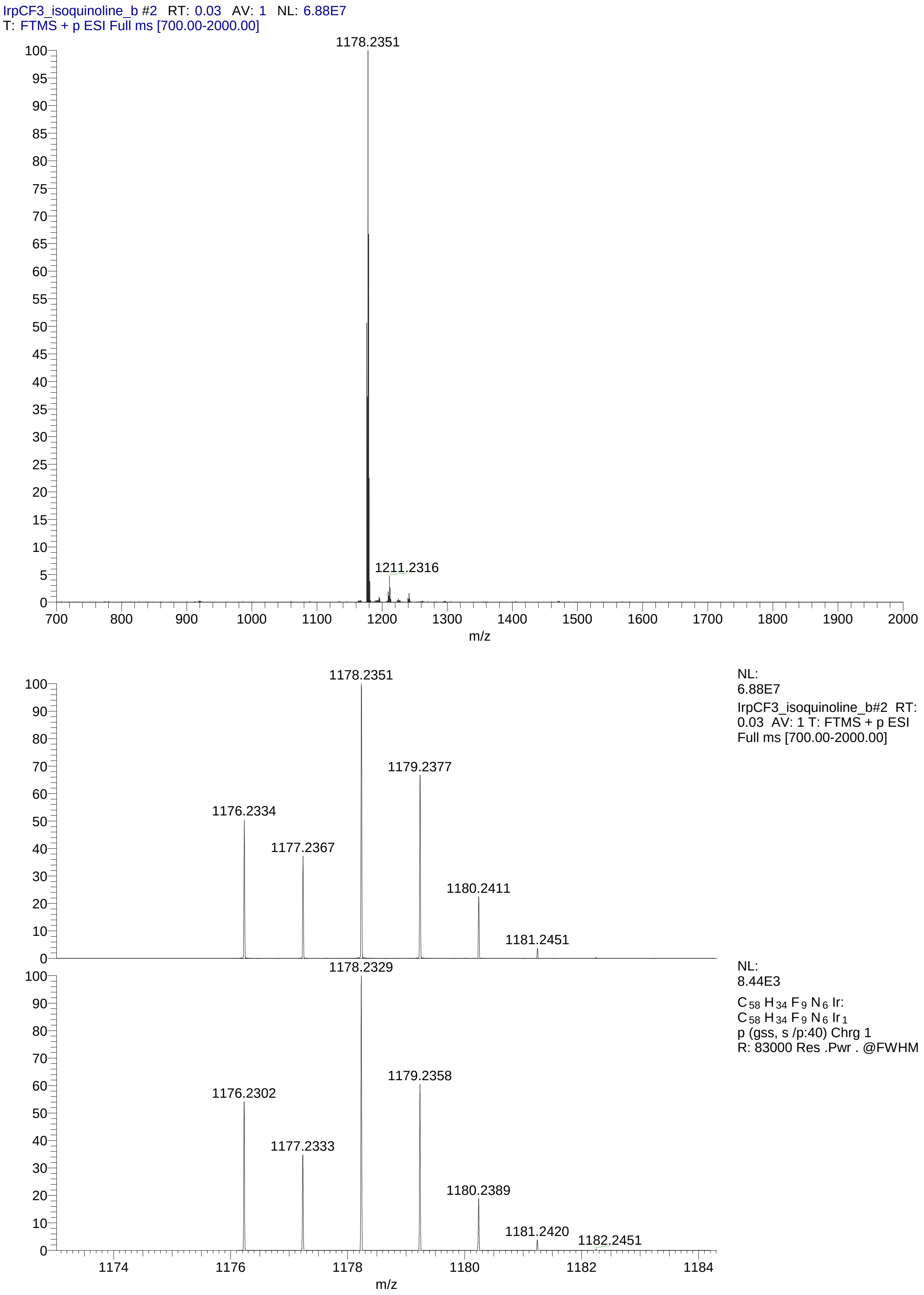

Figure 20. Electrospray ionization mass spectrum of Ir[T*p*CF_3PC]isoq$_2$.

C. ^{1}H NMR spectra

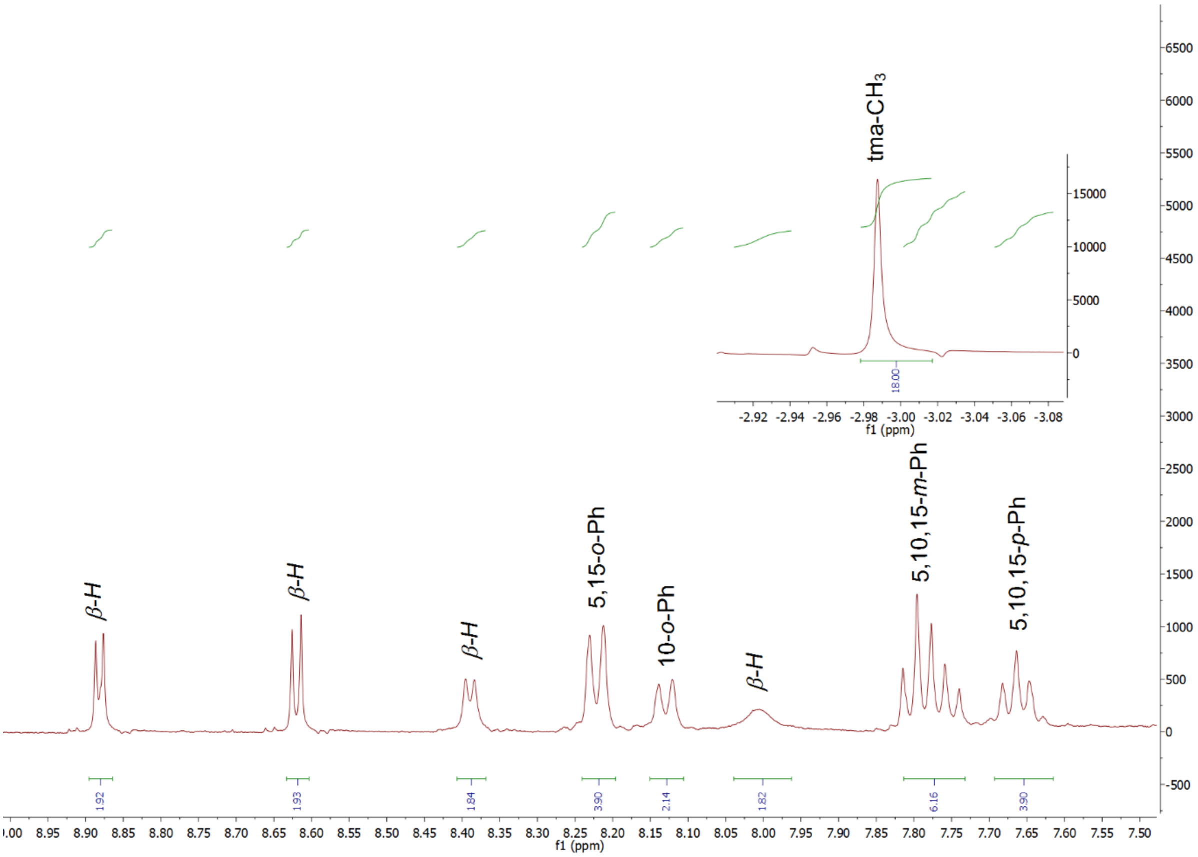

Figure 21. ^{1}H NMR spectrum of Ir[TPC]tma$_2$ in acetone-d_6.

(a)

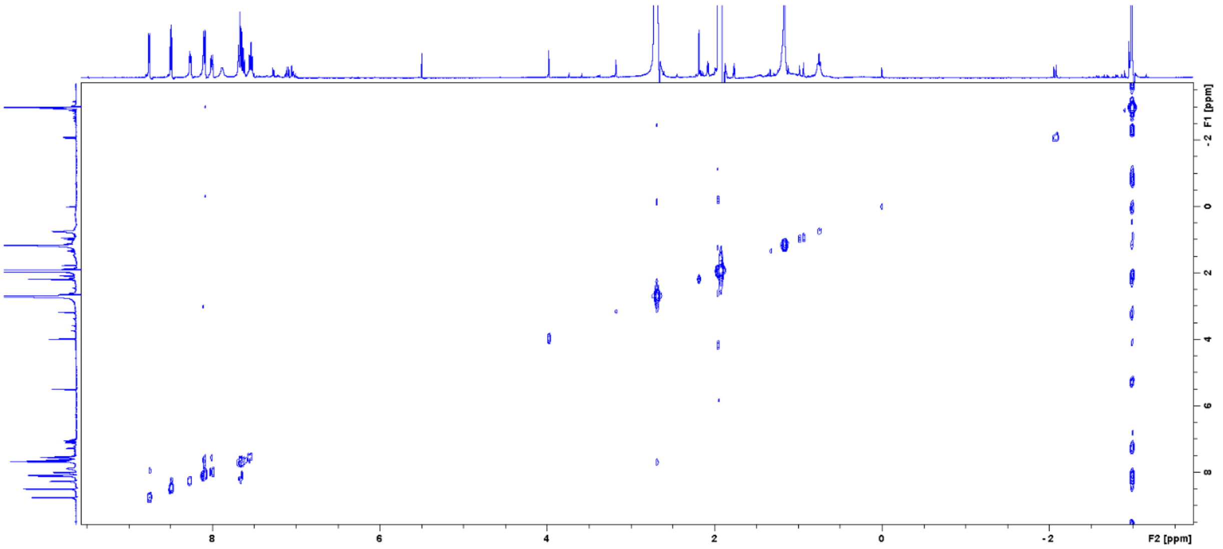

(b)

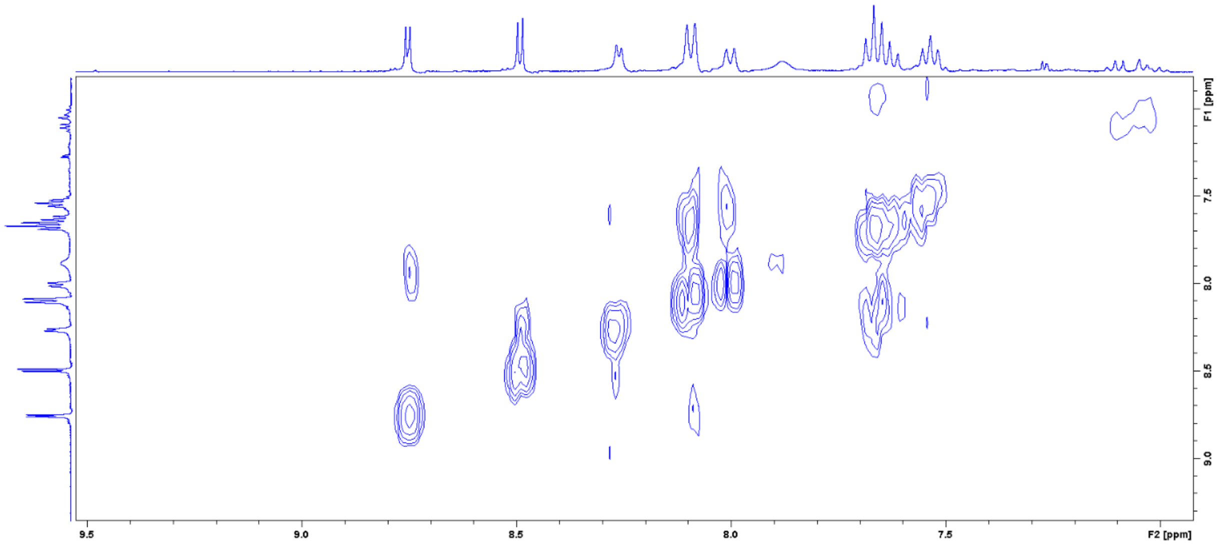

Figure 22. (a) COSY of Ir[TPC]tma$_2$ in acetone-d_6. **(b)** Close-up of aromatic area.

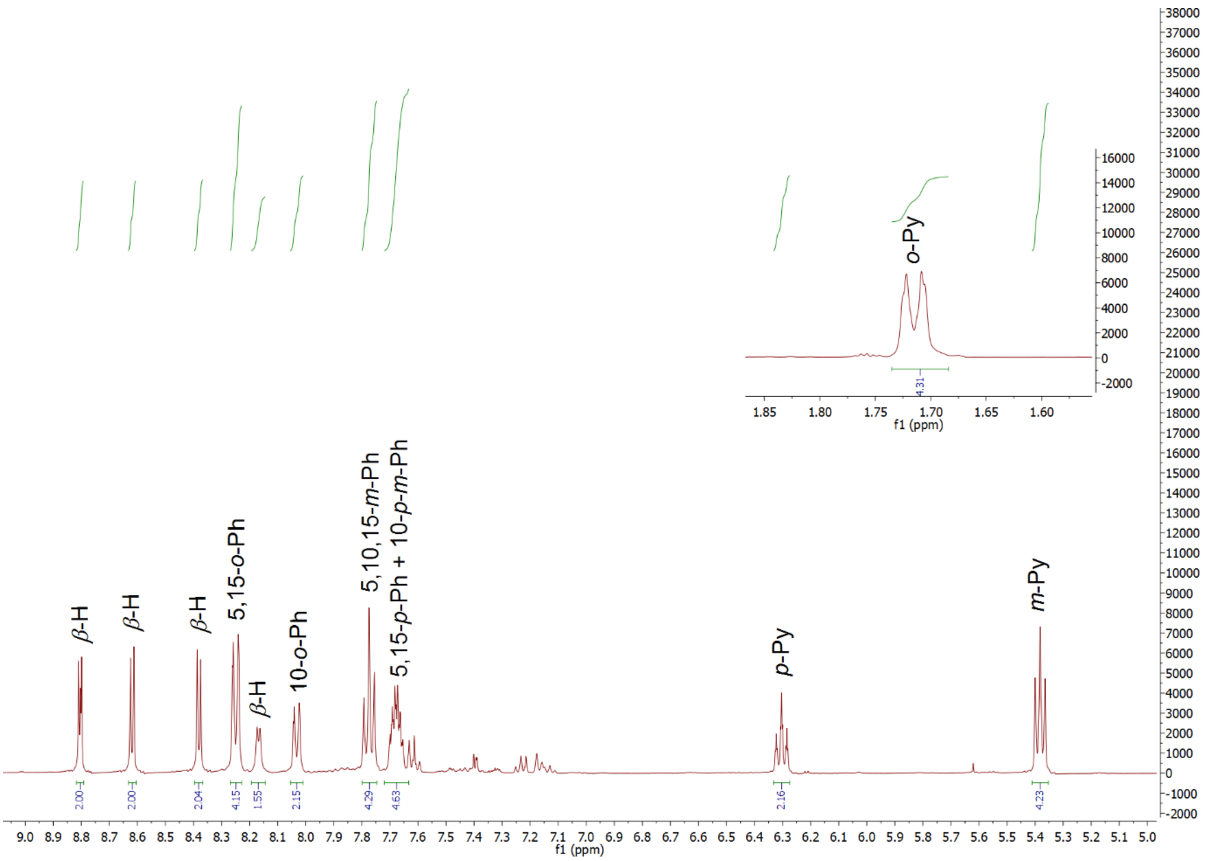

Figure 23. ^{1}H NMR spectrum of Ir[TPC]py$_2$ in acetone-d_6.

(a)

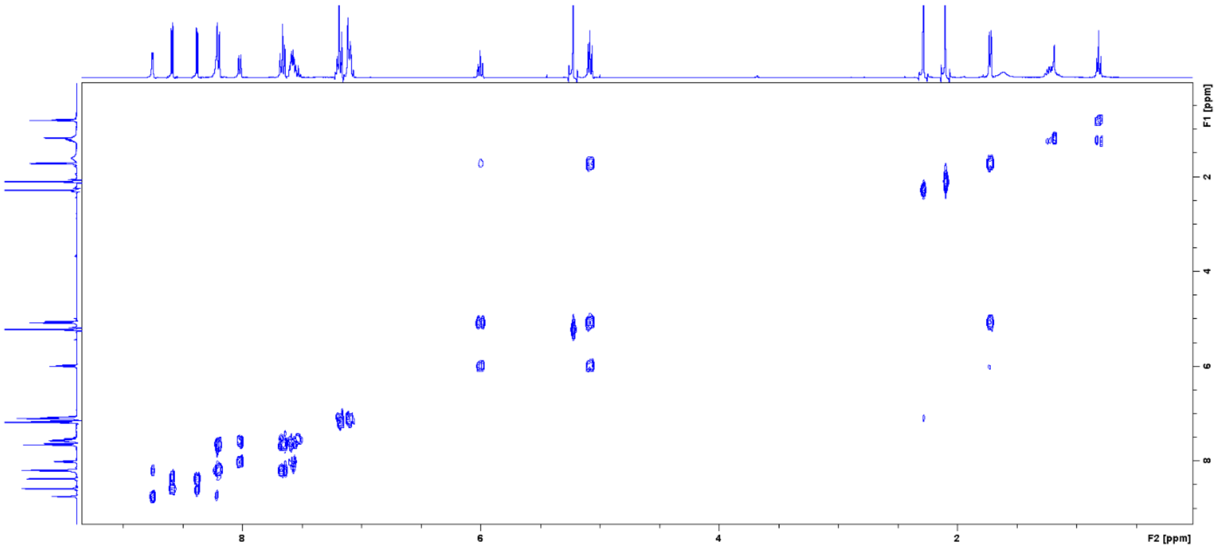

(b)

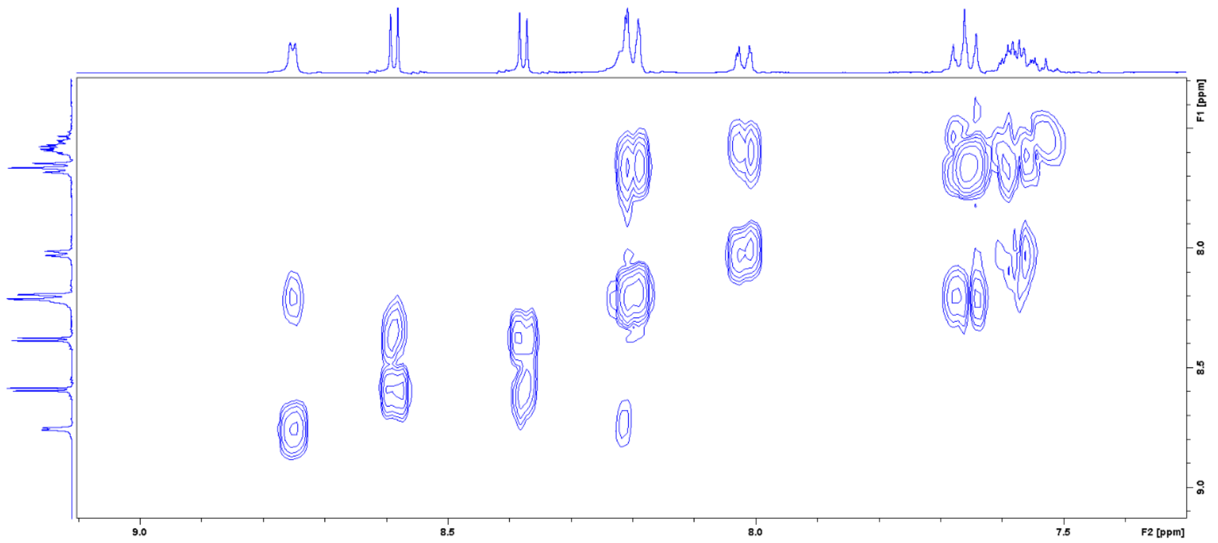

Figure 24. (a) COSY of Ir[TPC]py$_2$ in acetone-d_6. **(b)** Close-up of aromatic area.

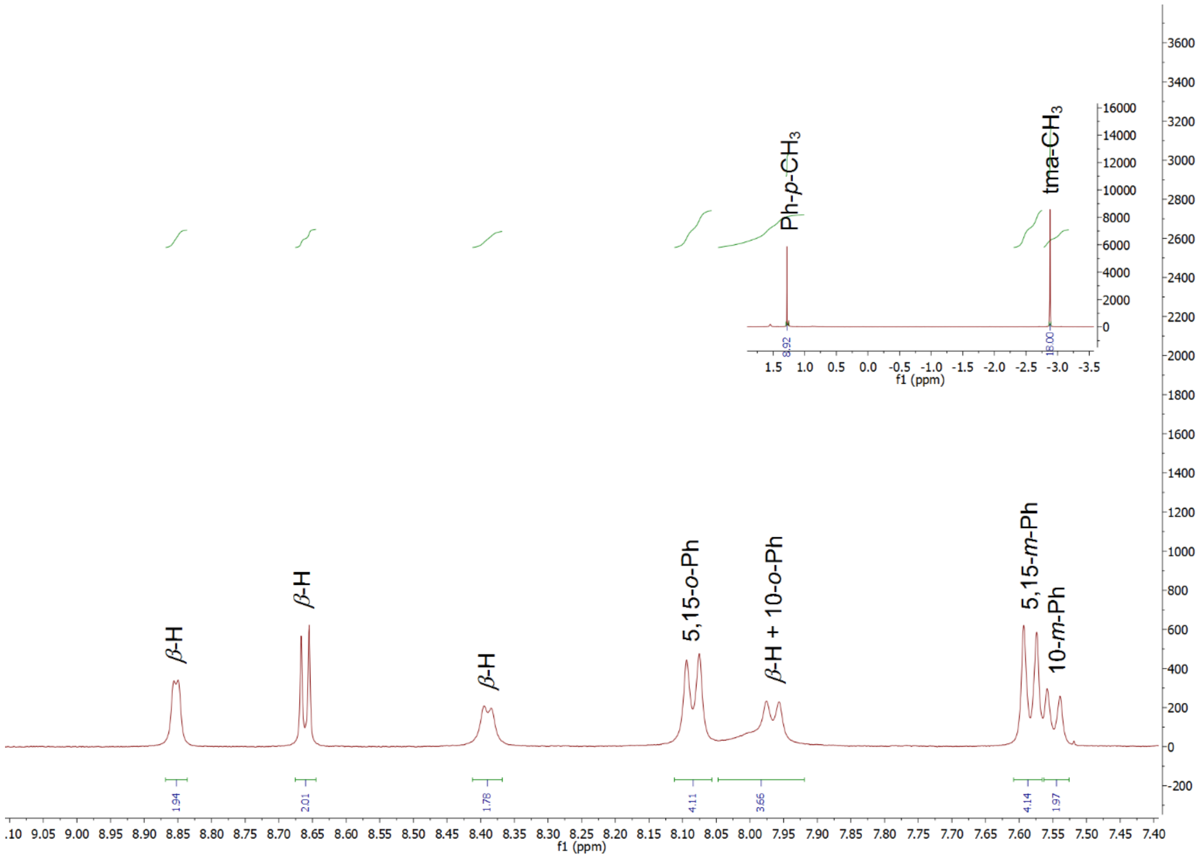

Figure 25. ^{1}H NMR spectrum of Ir[T*p*MePC]tma$_2$ in chloroform-*d*.

(a)

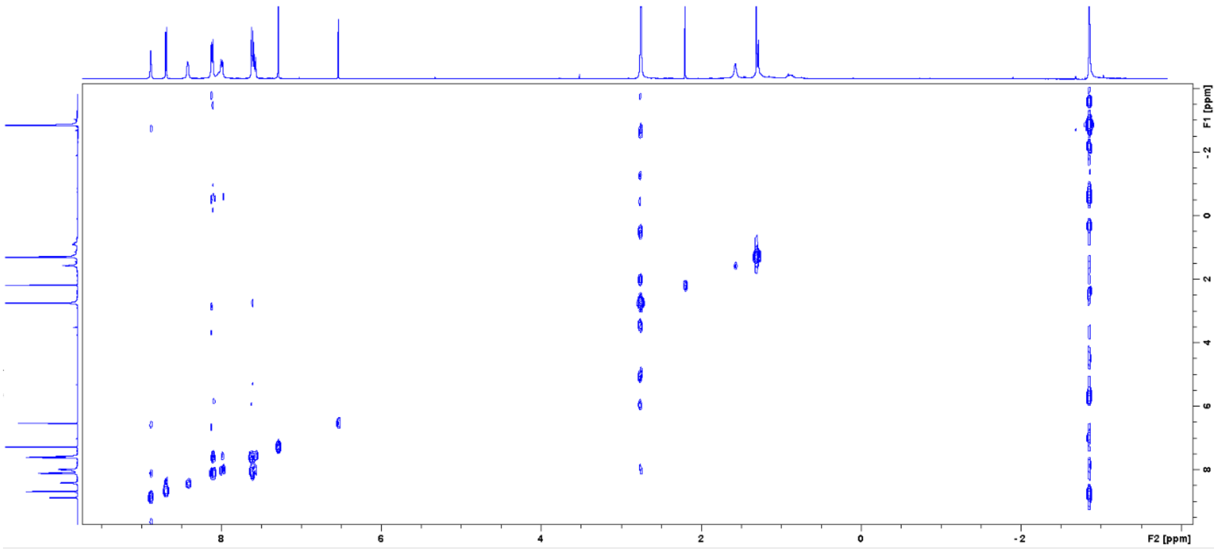

(b)

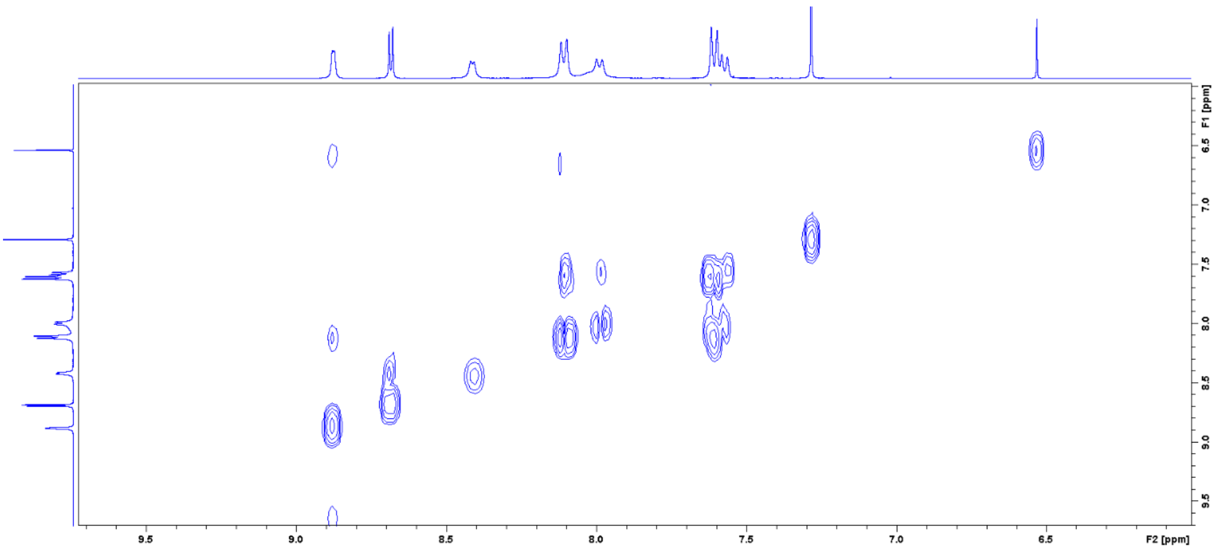

Figure 26. (a) COSY of Ir[T*p*MePC]tma$_2$ in chloroform-*d*. **(b)** Close-up of aromatic area.

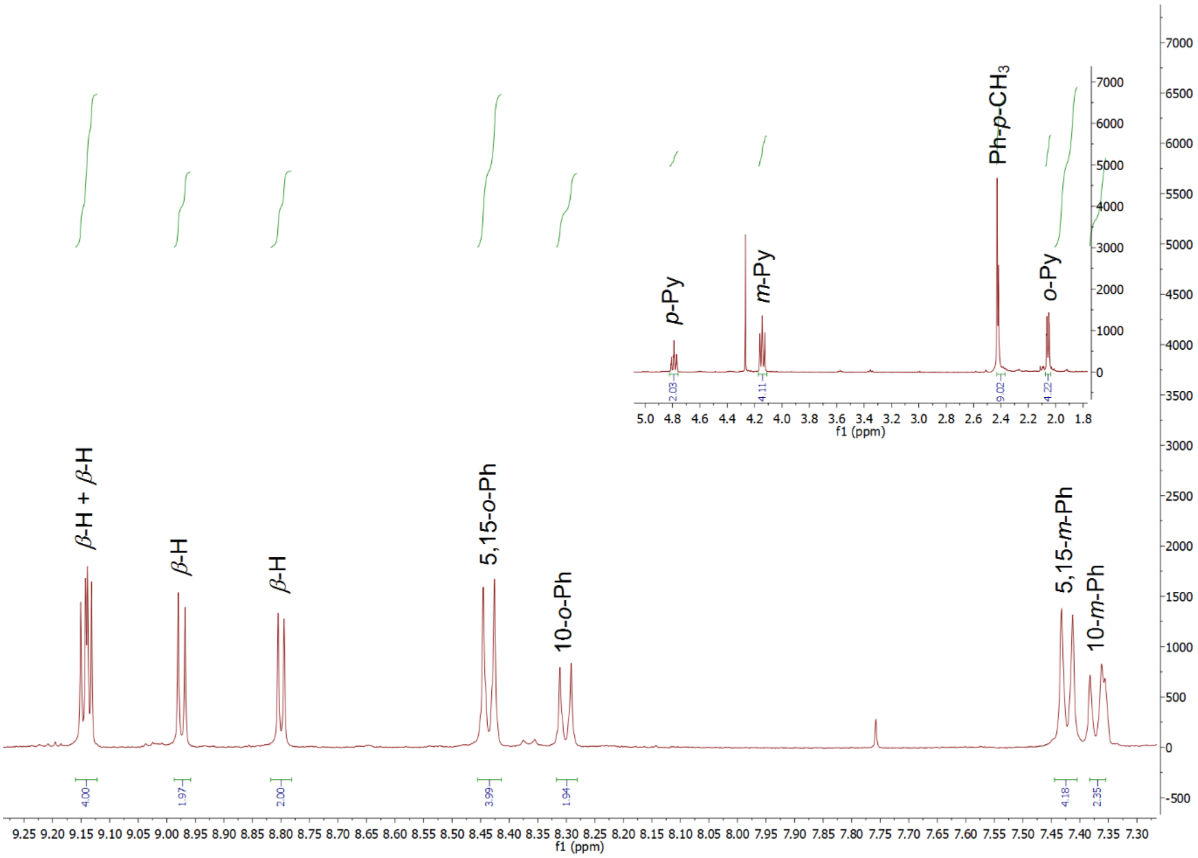

Figure 27. ^{1}H NMR spectrum of Ir[T*p*MePC]py$_2$ in benzene-*d*$_6$.

(a)

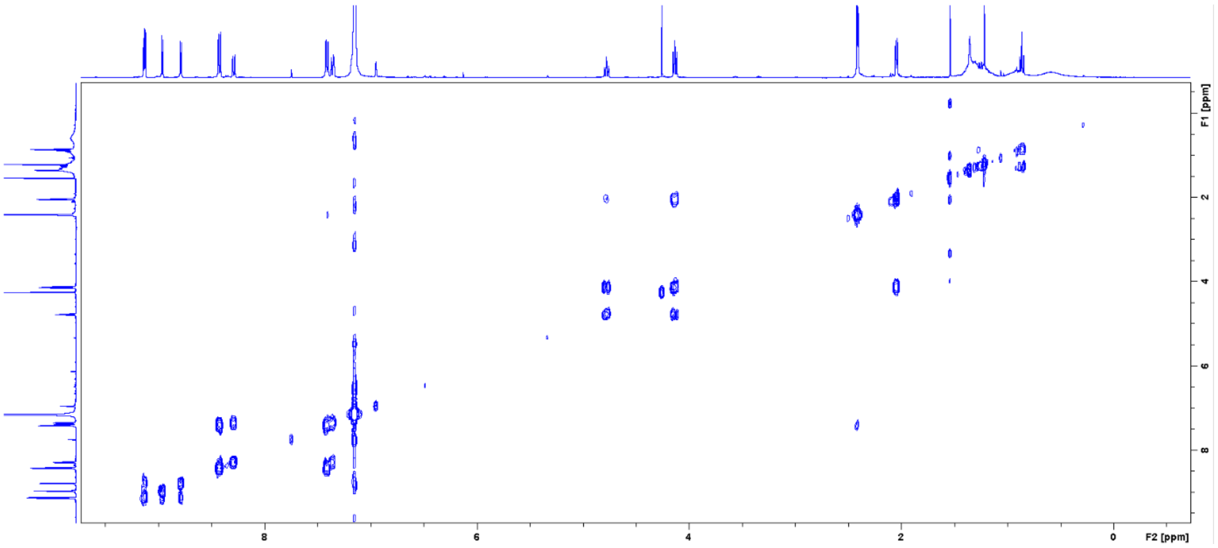

(b)

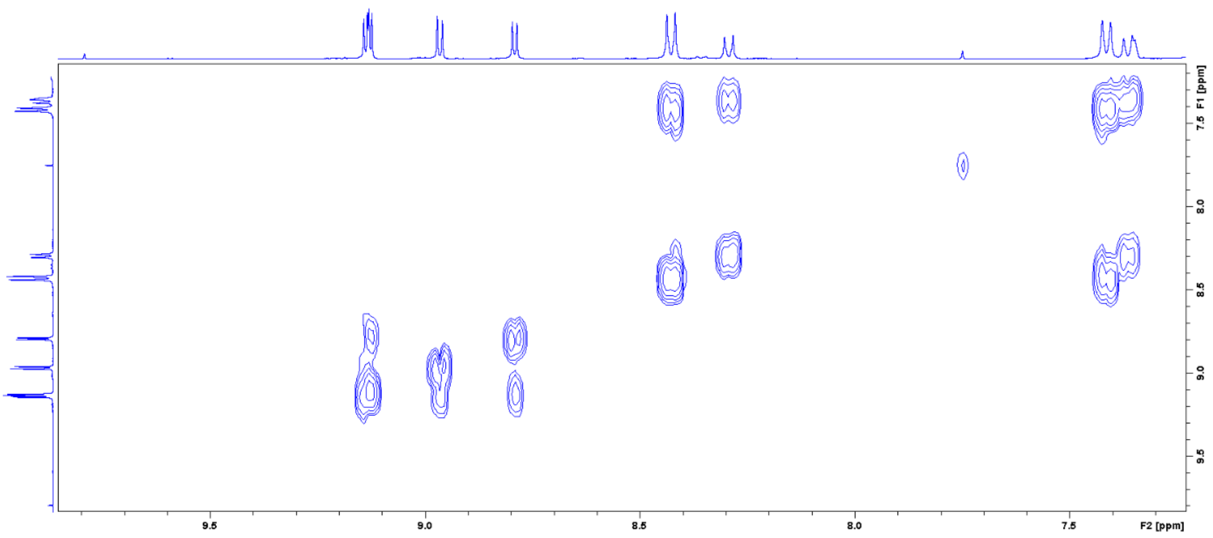

Figure 28. (a) COSY of Ir[T*p*MePC]py$_2$ in benzene-d_6. **(b)** Close-up of aromatic area.

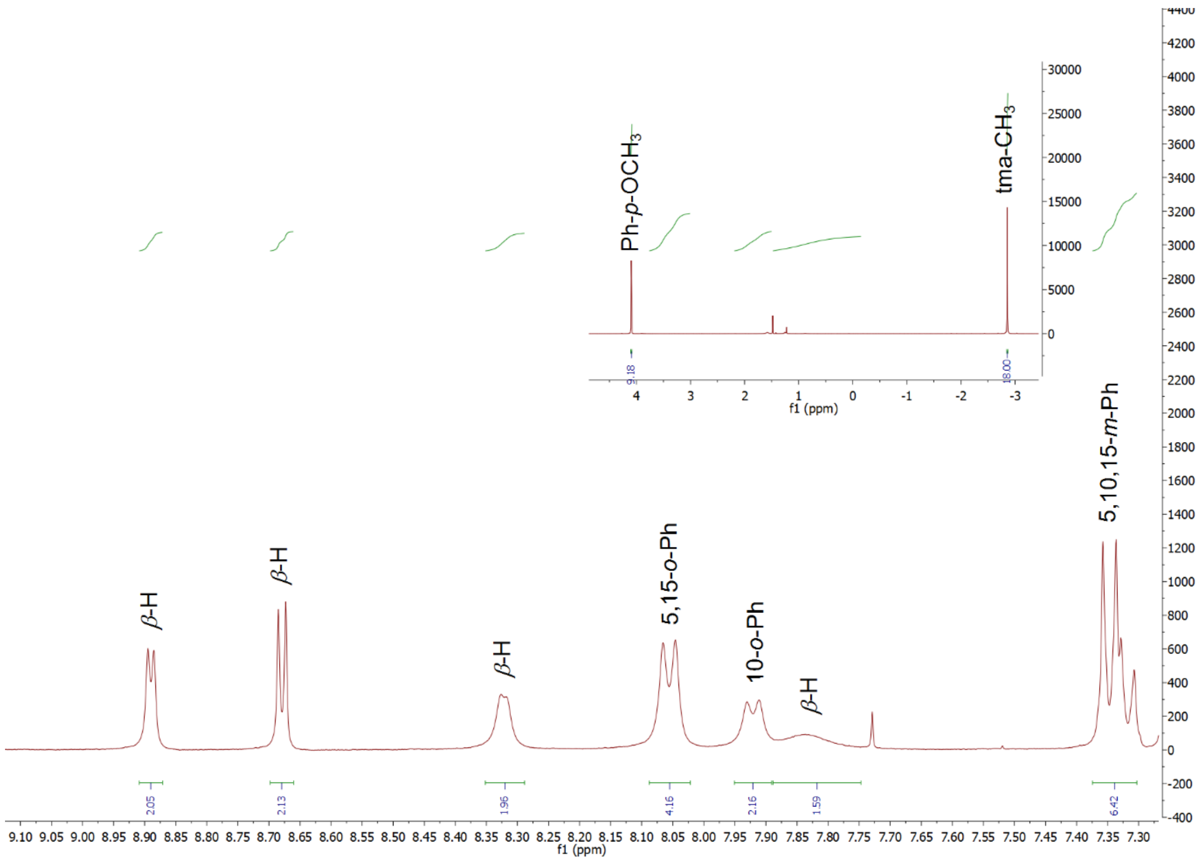

Figure 29. ^{1}H NMR spectrum of Ir[T*p*OMePC]tma$_2$ in chloroform-*d*.

(a)

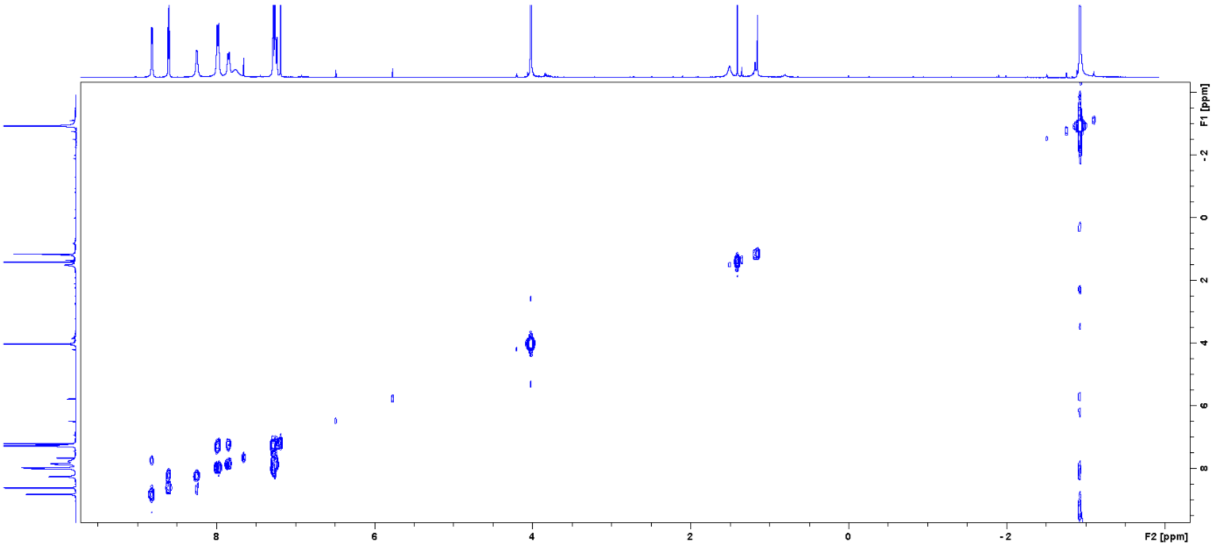

(b)

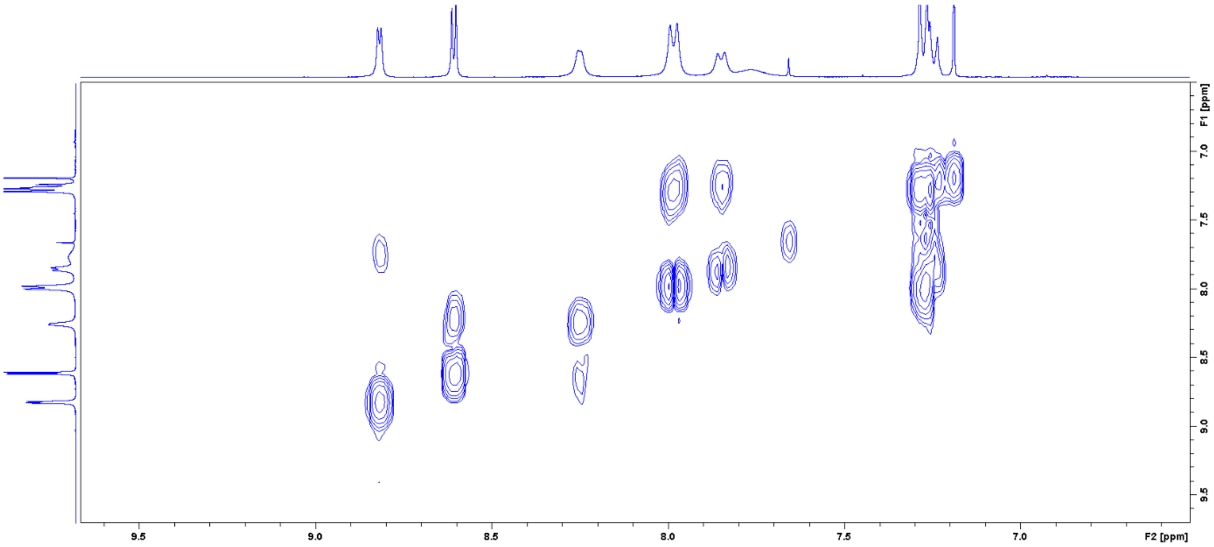

Figure 30. (a) COSY of Ir[T*p*OMePC]tma$_2$ in chloroform-*d*. **(b)** Close-up of aromatic area.

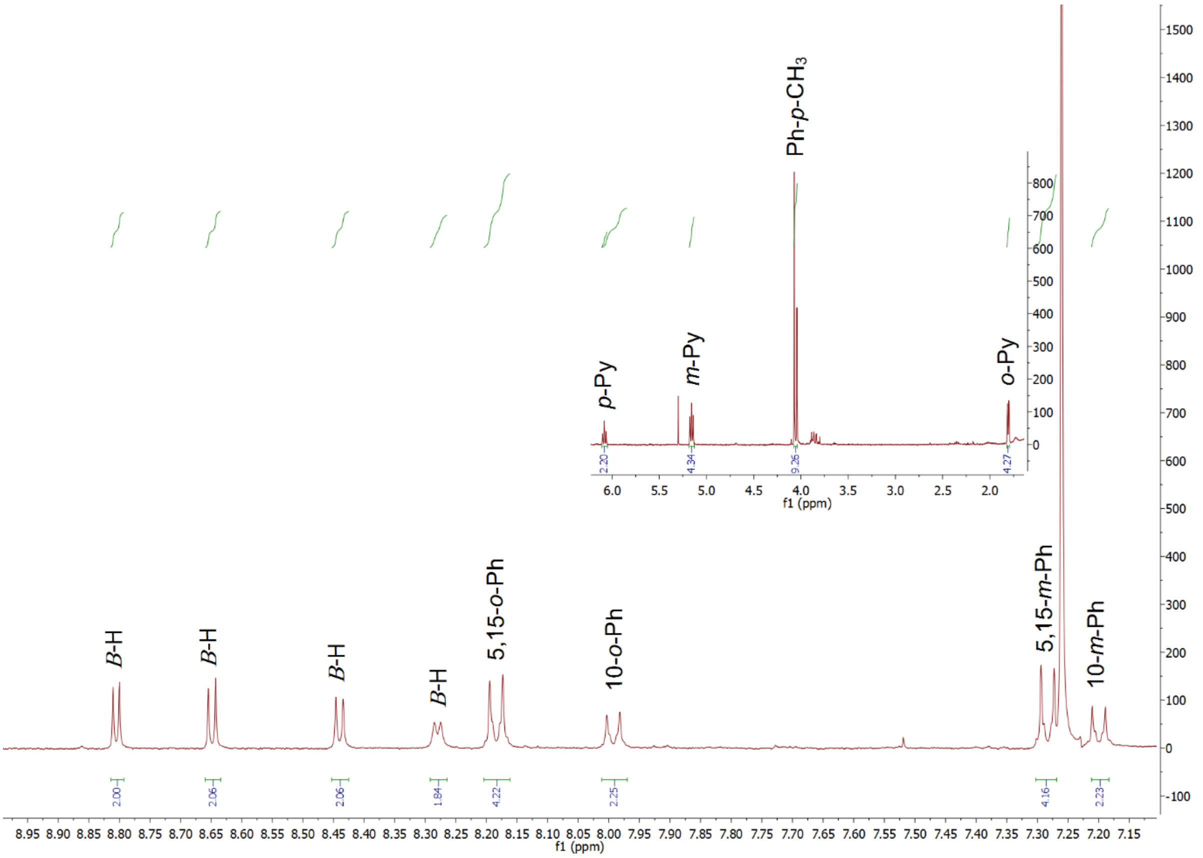

Figure 31. ^{1}H NMR spectrum of Ir[T*p*OMePC]py$_2$ in benzene-*d*$_6$.

(a)

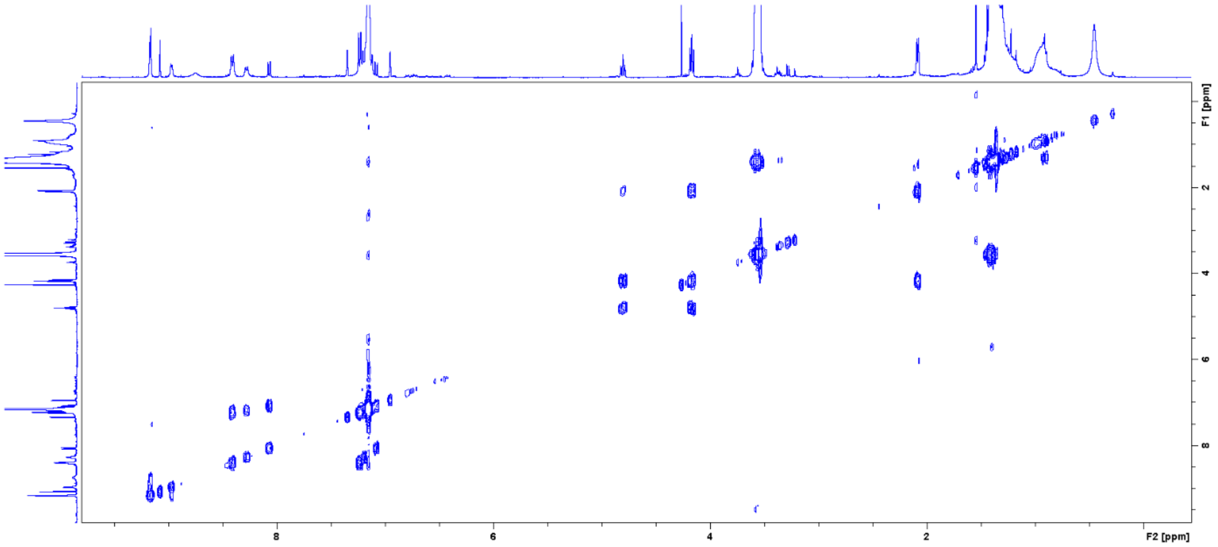

(b)

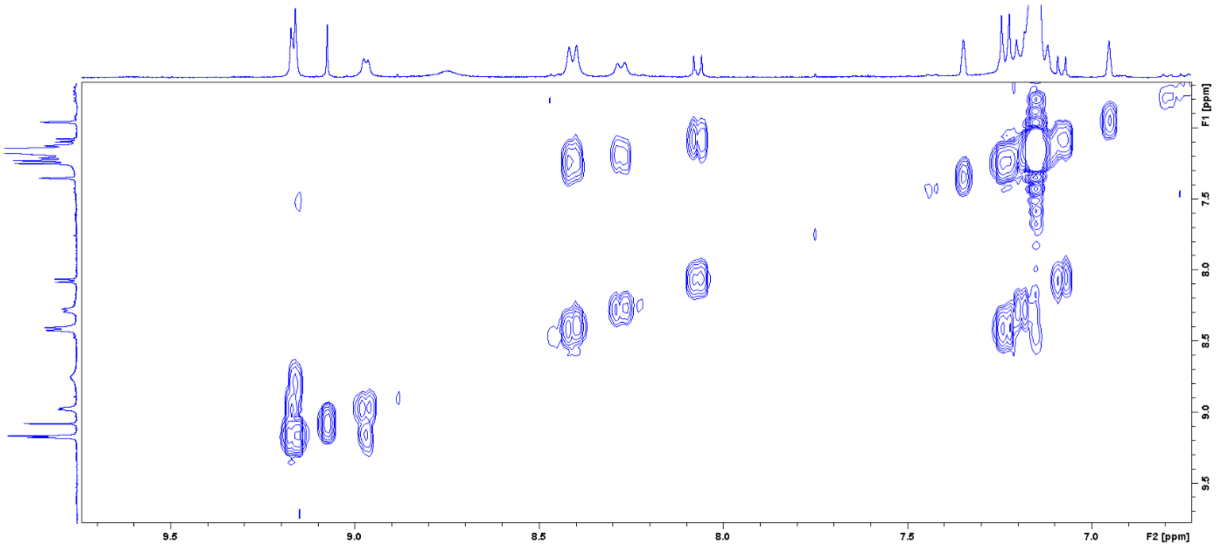

Figure 32. (a) COSY of Ir[T*p*OMePC]py$_2$ in benzene-*d*$_6$. **(b)** Close-up of aromatic area.

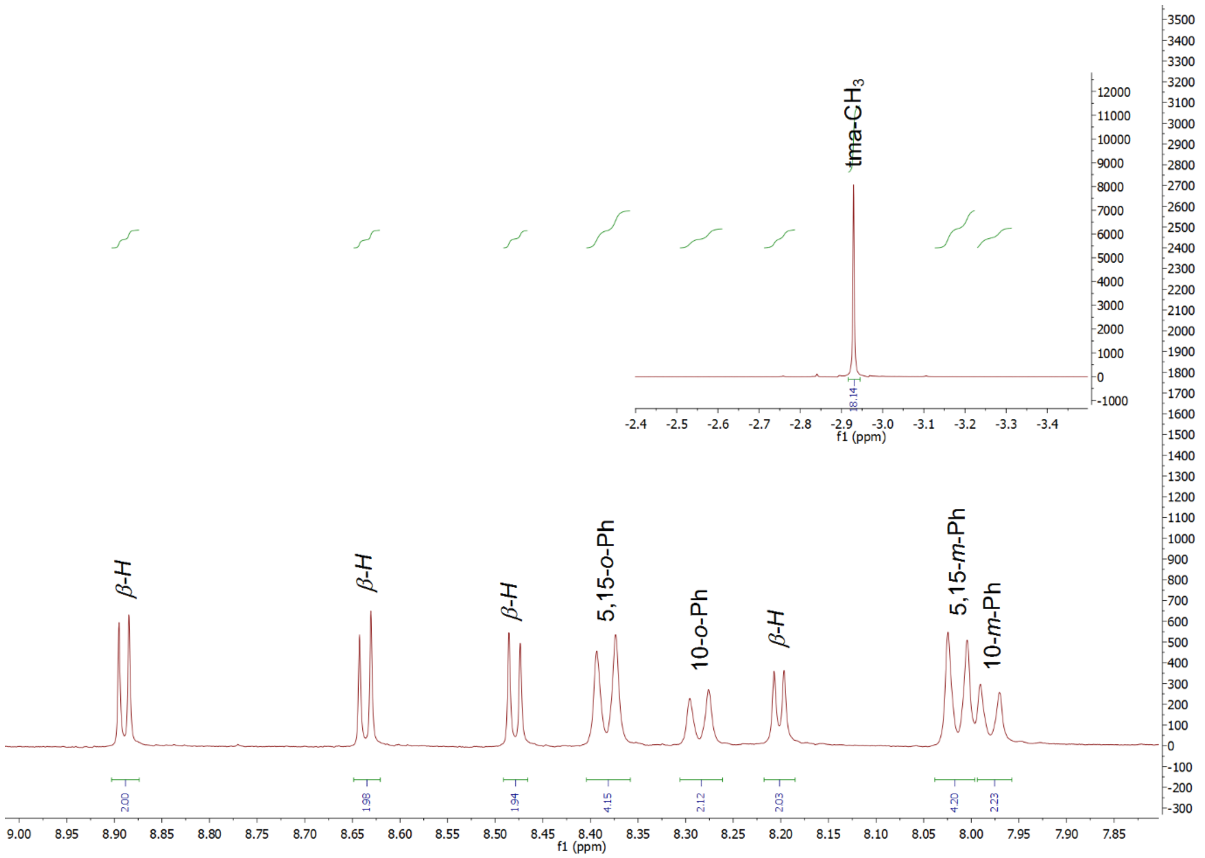

Figure 33. ^{1}H NMR spectrum of Ir[T*p*CF$_3$PC]tma$_2$ in chloroform-*d*.

(a)

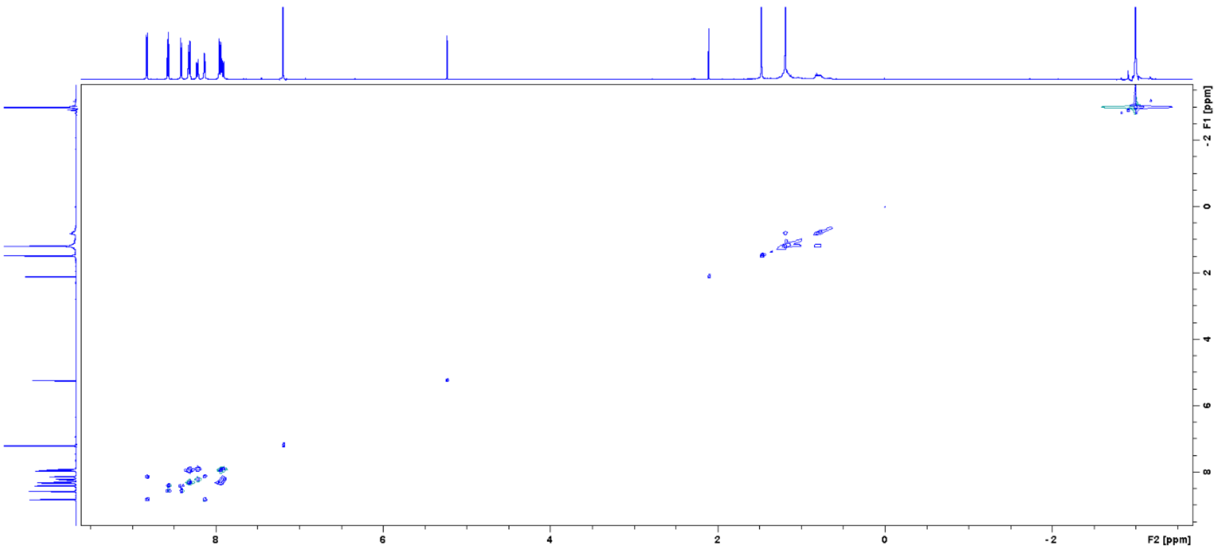

(b)

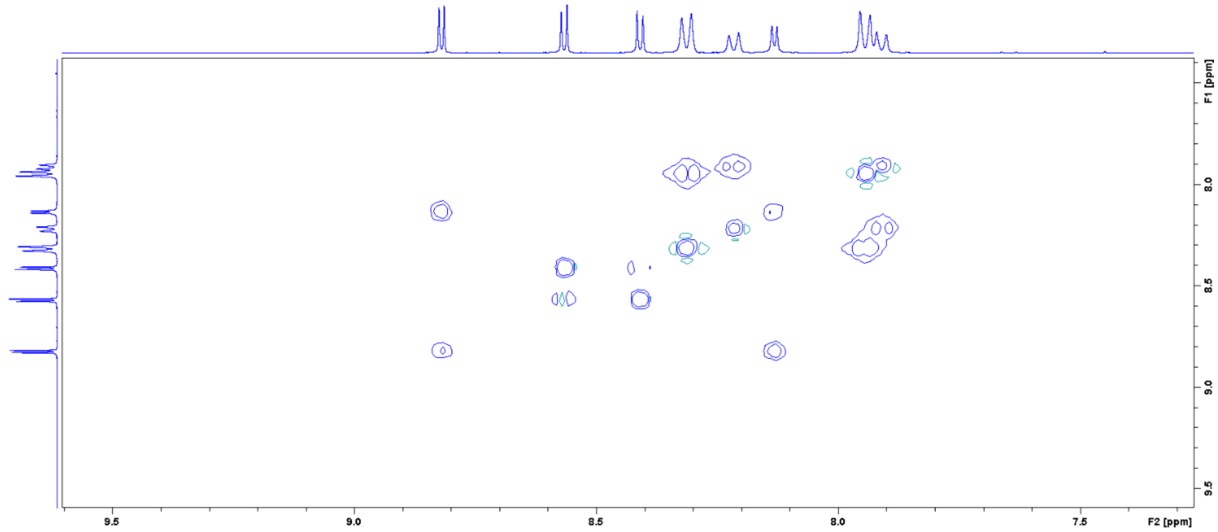

Figure 34. (a) COSY of Ir[T*p*CF_3PC]tma_2 in chloroform-*d*. **(b)** Close-up of aromatic area.

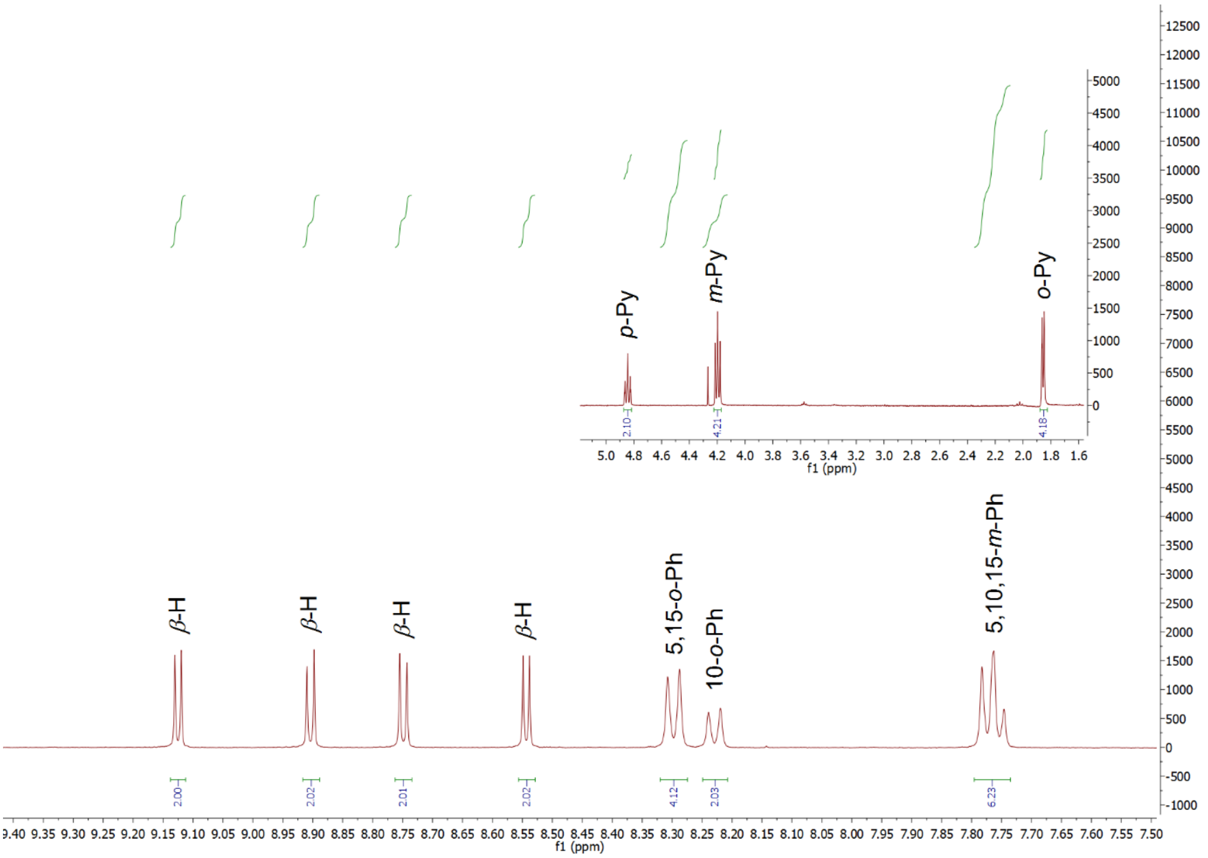

Figure 35. ^{1}H NMR spectrum of Ir[T*p*CF_3PC]py_2 in benzene-d_6.

(a)

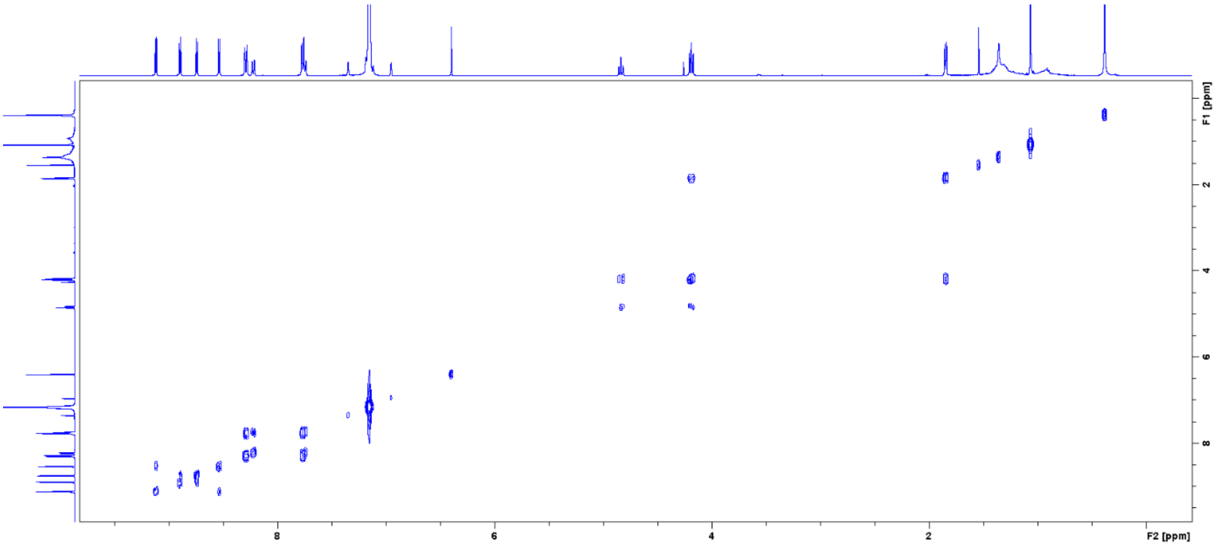

(b)

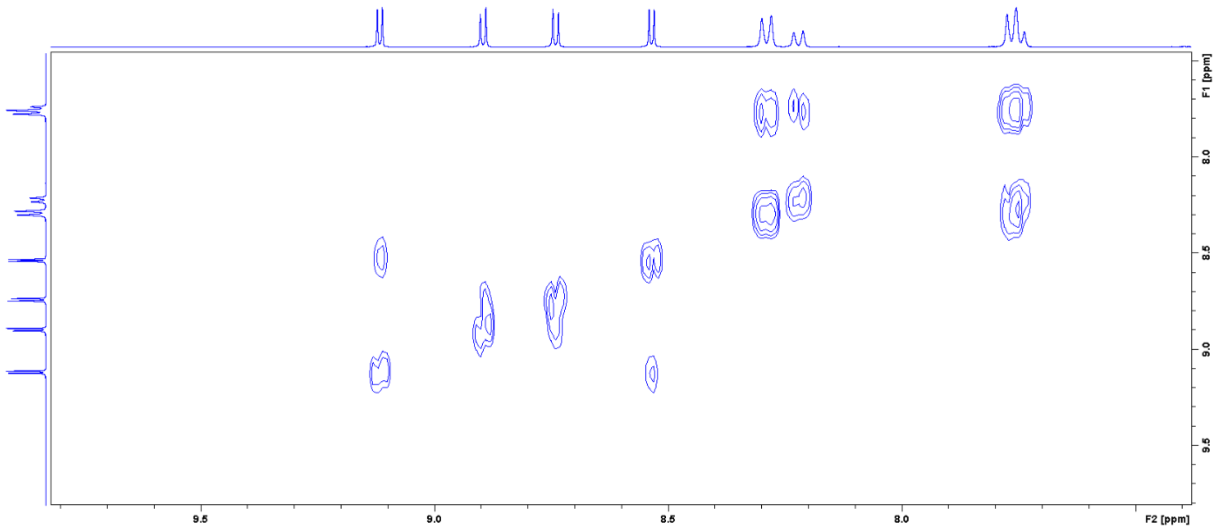

Figure 36. (a) COSY of Ir[T*p*CF_3PC]py_2 in benzene-*d*6. **(b)** Close-up of aromatic area.

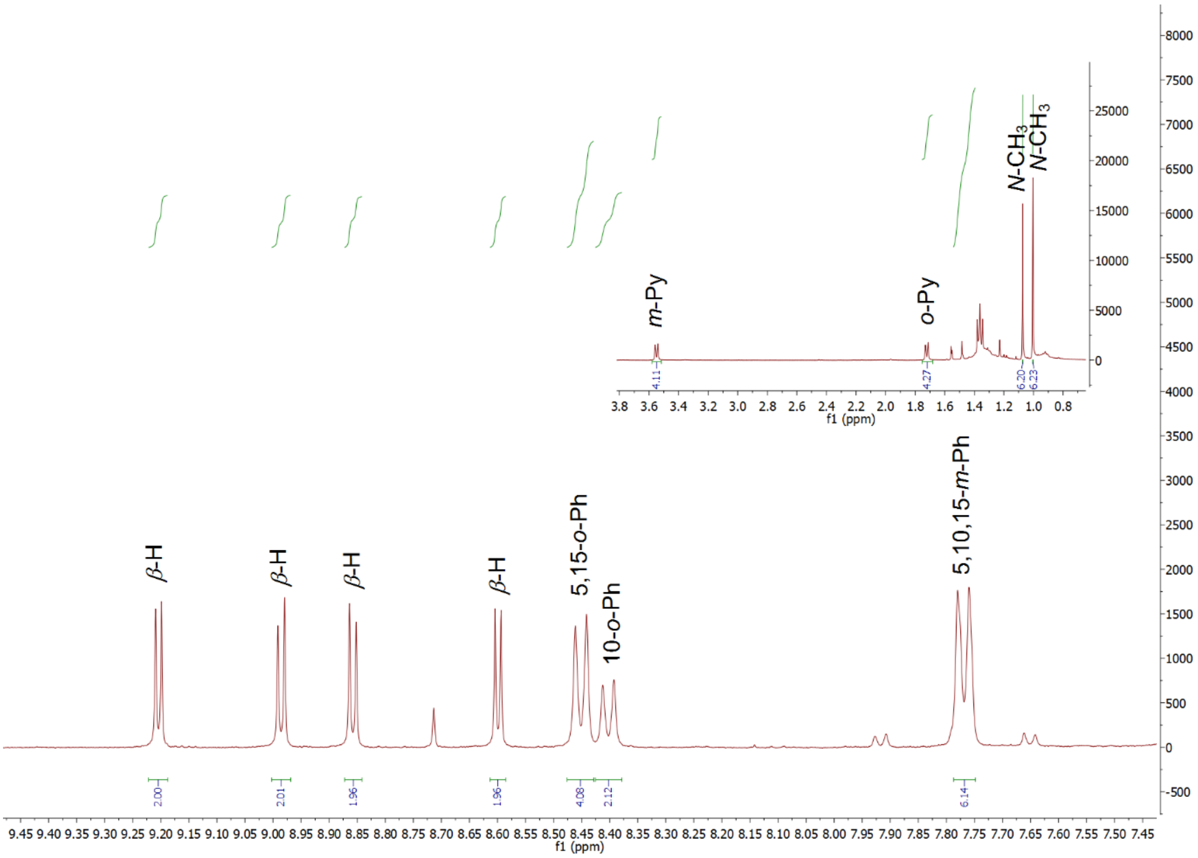

Figure 37. ^{1}H NMR spectrum of Ir[T*p*CF$_3$PC]dmap$_2$ in benzene-d_6.

(a)

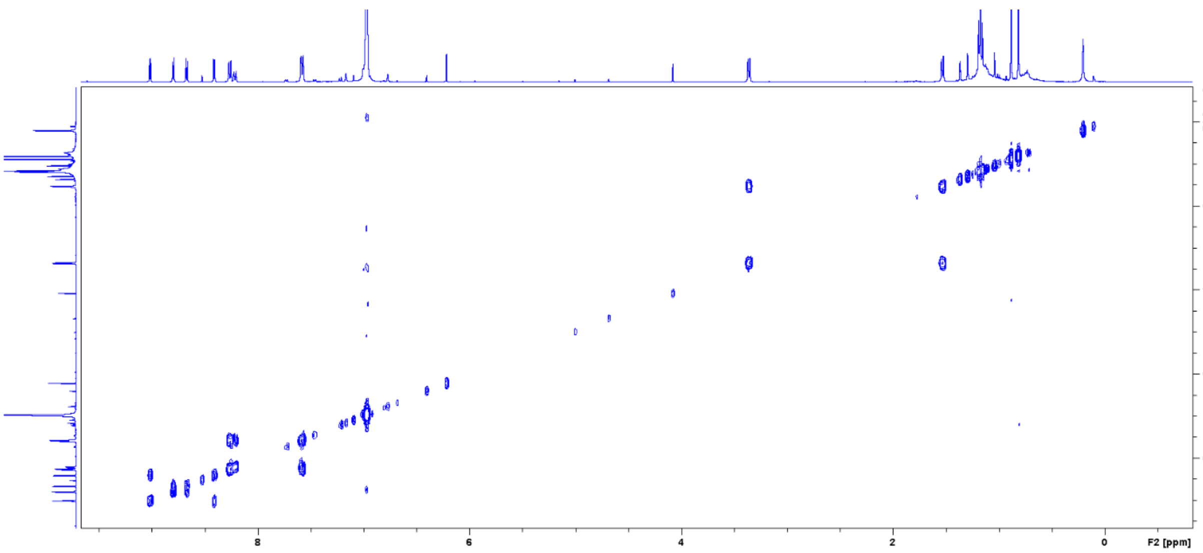

(b)

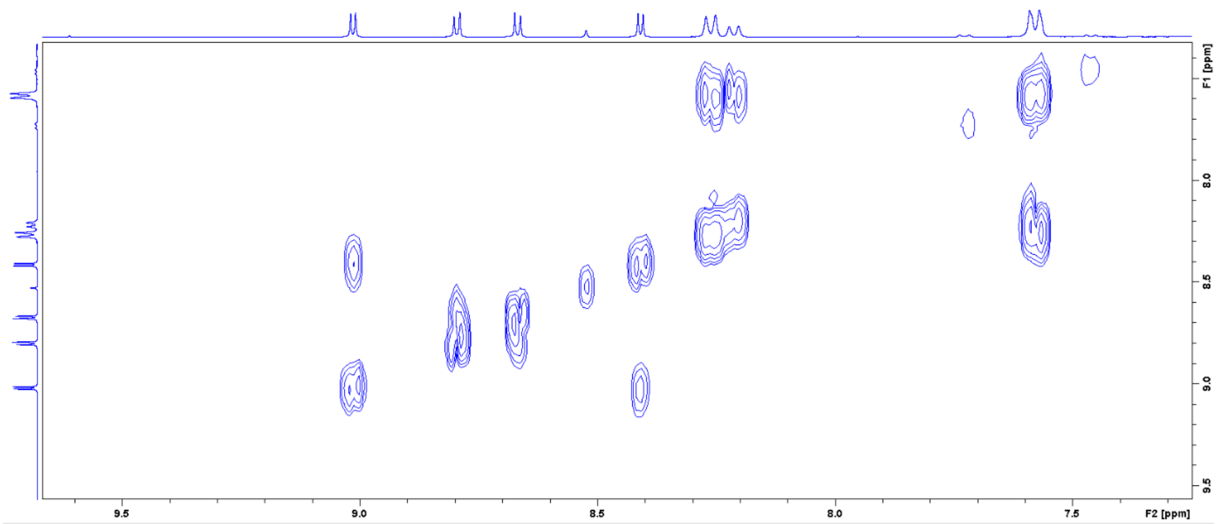

Figure 38. (a) COSY of Ir[T*p*CF$_3$PC]dmap$_2$ in benzene-*d*$_6$. **(b)** Close-up of aromatic area.

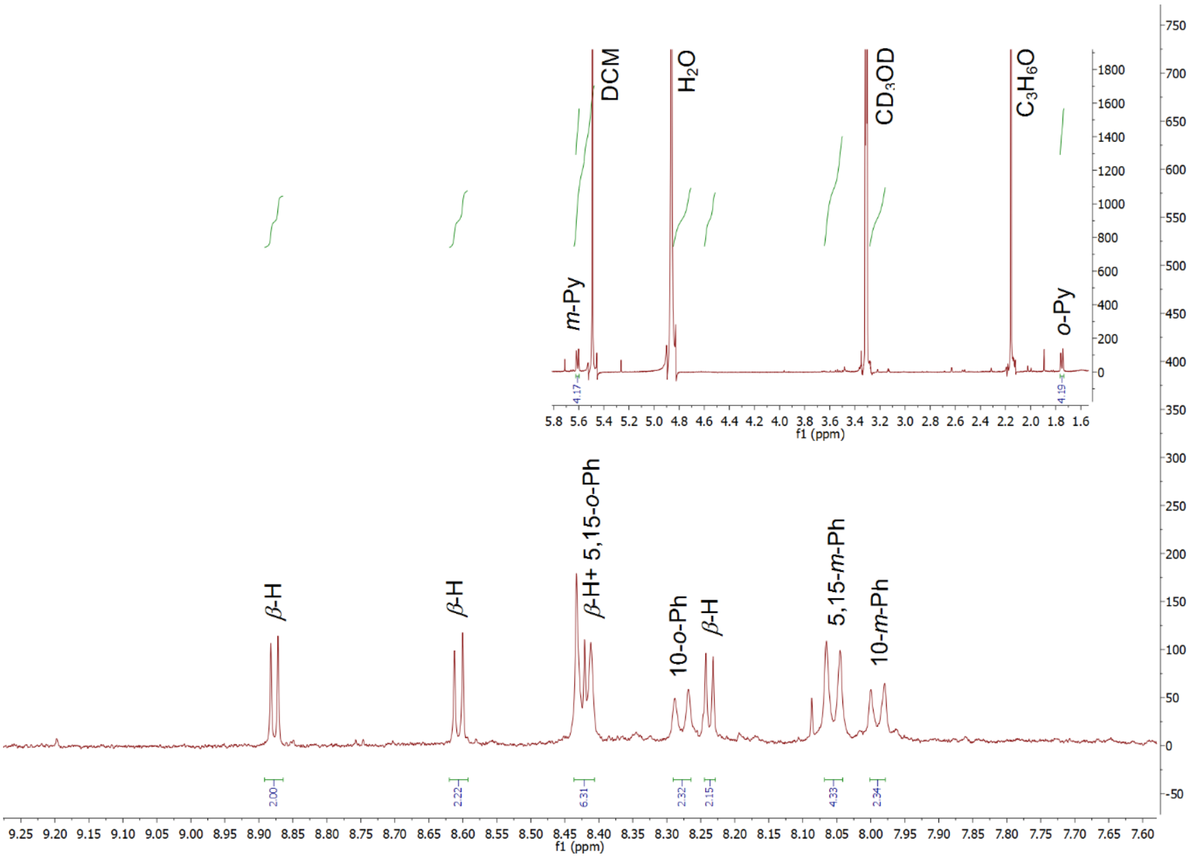

Figure 39. ^{1}H NMR spectrum of Ir[T*p*CF_3PC]4pa_2 in methanol-d_4.

(a)

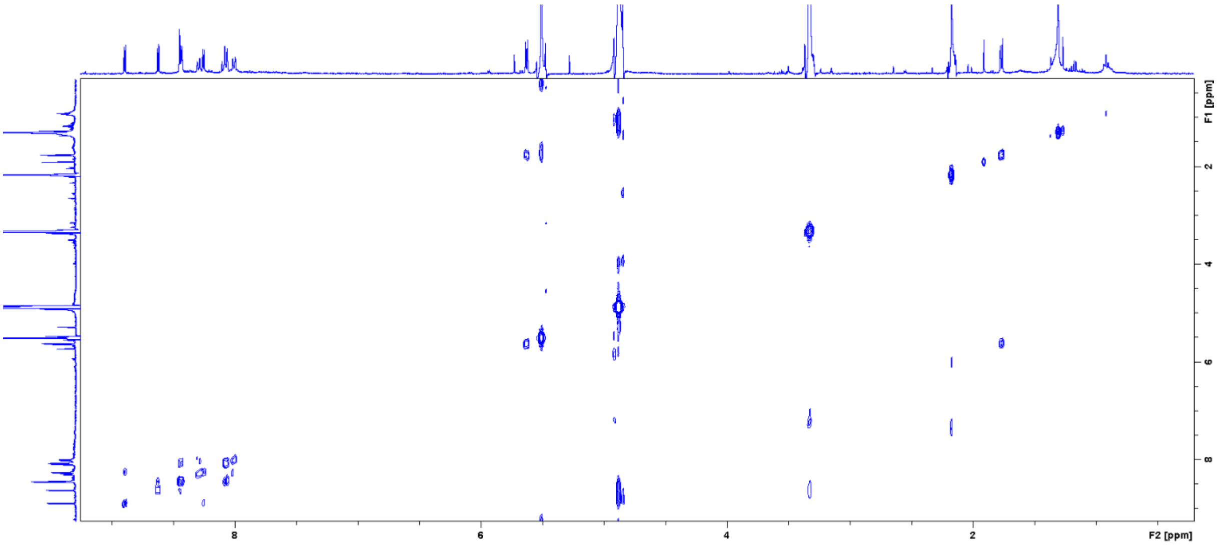

(b)

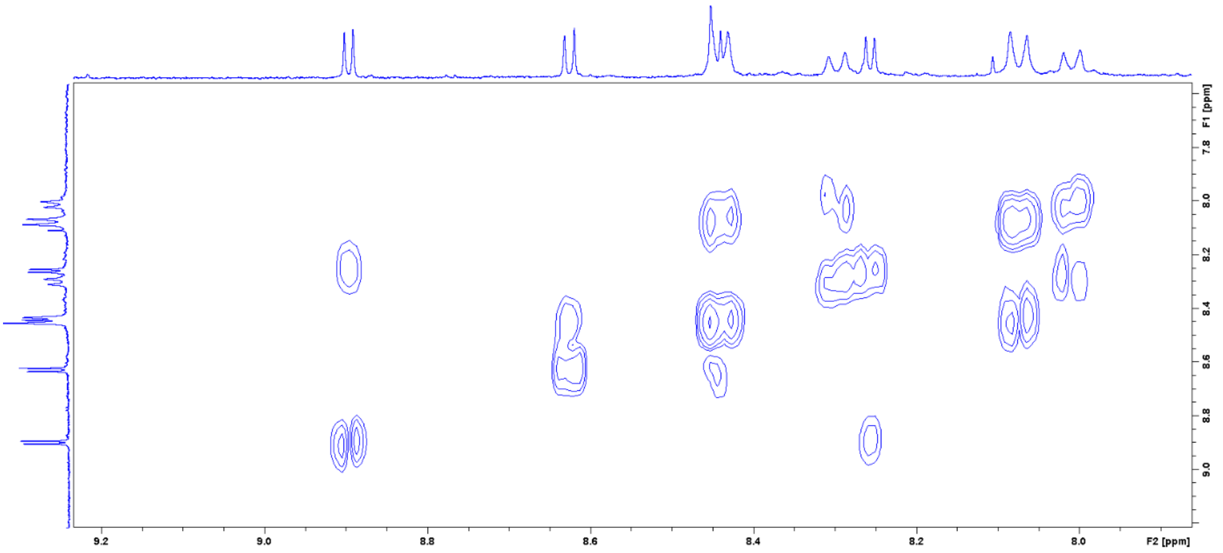

Figure 40. (a) COSY of Ir[T*p*CF_3PC]4pa_2 in methanol-d_4. **(b)** Close-up of aromatic area.

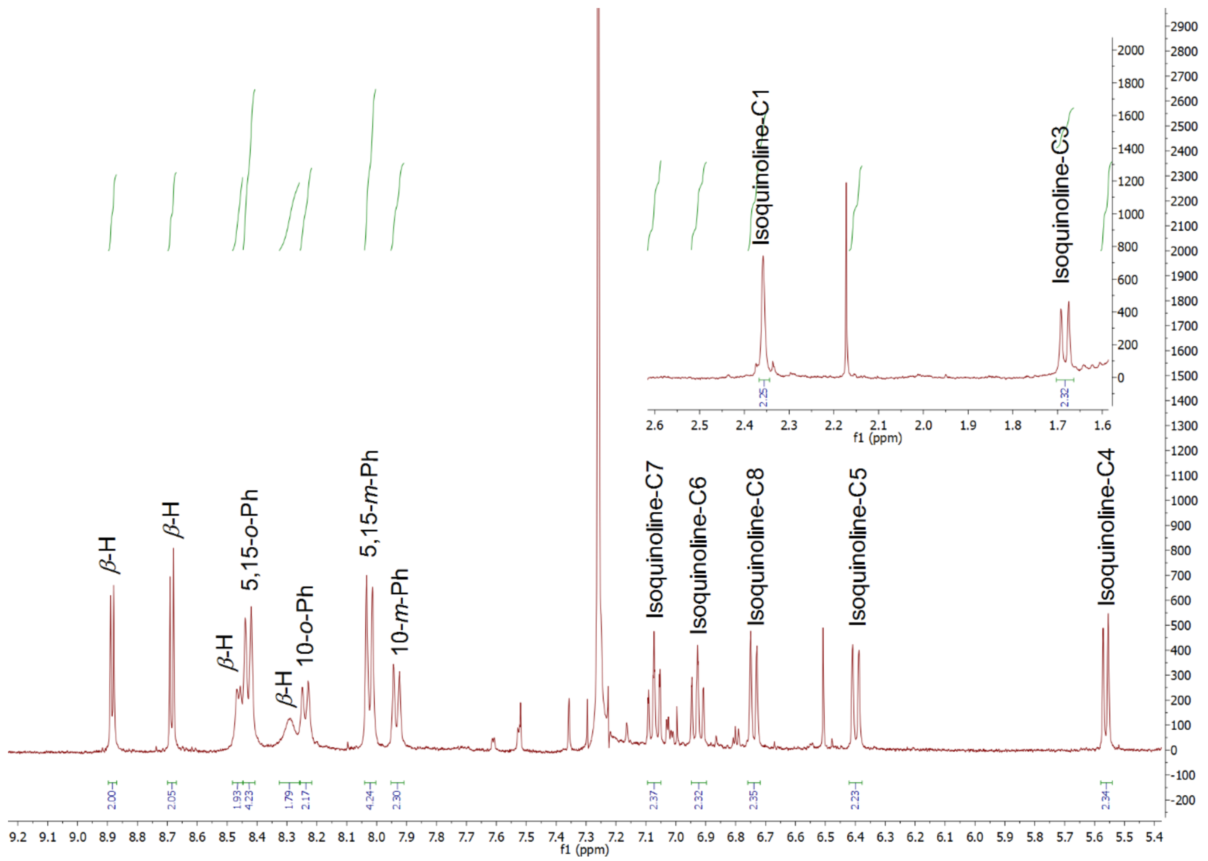

Figure 41. ^{1}H NMR spectrum of Ir[T*p*CF$_3$PC]isoq$_2$ in chloroform-*d*.

(a)

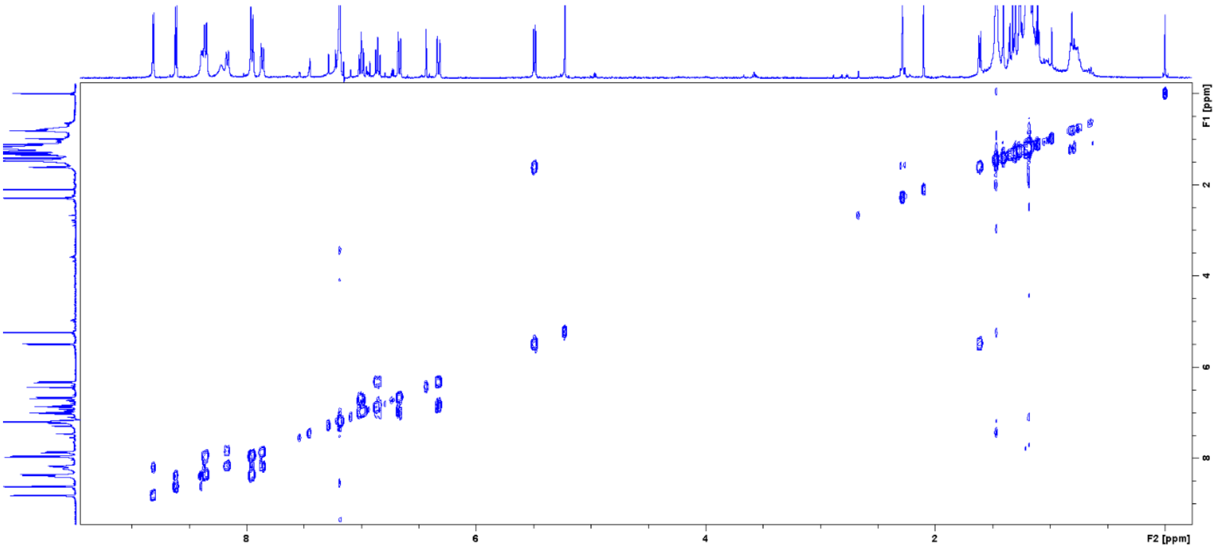

(b)

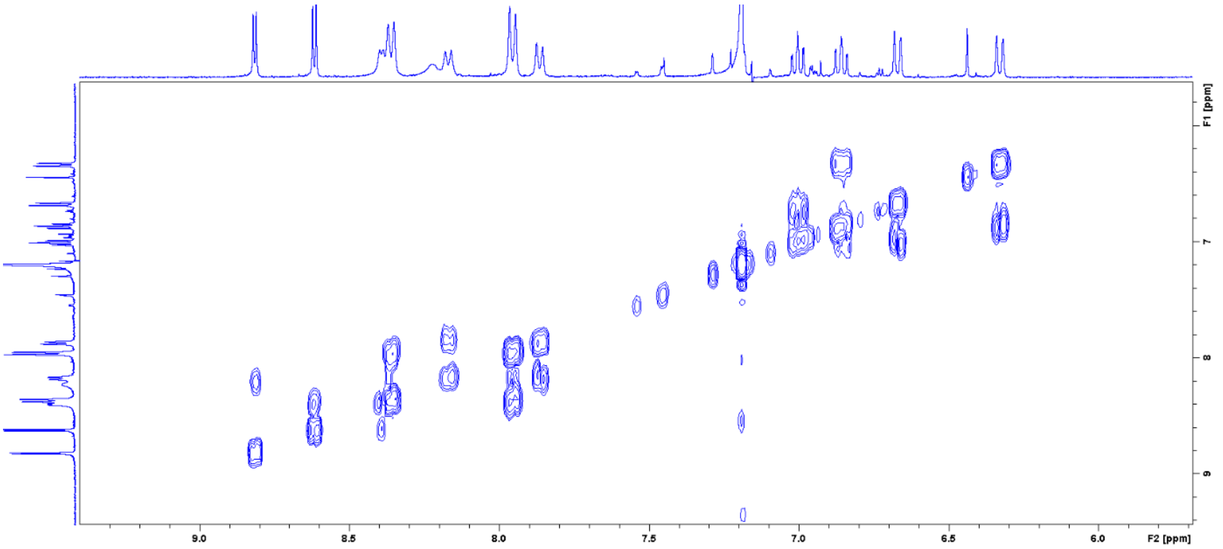

Figure 42. (a) COSY of Ir[T*p*CF_3PC]isoq$_2$ in chloroform-*d*. **(b)** Close-up of aromatic area.
